# Noble 3,4-Seco-triterpenoid Glycosides from the Fruits of *Acanthopanax sessiliflorus* and Their Anti-Neuroinflammatory Effects

**DOI:** 10.3390/antiox10091334

**Published:** 2021-08-24

**Authors:** Bo-Ram Choi, Hyoung-Geun Kim, Wonmin Ko, Linsha Dong, Dahye Yoon, Seon Min Oh, Young-Seob Lee, Dong-Sung Lee, Nam-In Baek, Dae Young Lee

**Affiliations:** 1 Department of Herbal Crop Research, National Institute of Horticultural and Herbal Science, RDA, Eumseong 27709, Korea; bmcbr@korea.kr (B.-R.C.); dahyeyoon@korea.kr (D.Y.); seonmin88@korea.kr (S.M.O.); youngseoblee@korea.kr (Y.-S.L.); 2 Graduate School of Biotechnology and Department of Oriental Medicinal Biotechnology, Kyung Hee University, Yongin 17104, Korea; zwang05@khu.ac.kr (H.-G.K.); nibaek@khu.ac.kr (N.-I.B.); 3 College of Pharmacy, Chosun University, Gwangju 61452, Korea; rabis815@naver.com (W.K.); donglinsha011@163.com (L.D.); dslee2771@chosun.ac.kr (D.-S.L.)

**Keywords:** *Acanthopanax sessiliflorus* fruit, acanthosessiliosides, anti-inflammation, anti-neuroinflammation, NMR, quantification

## Abstract

*Acanthopanax sessiliflorus* (Araliaceae) have been reported to exhibit many pharmacological activities. Our preliminary study suggested that *A. sessiliflorus* fruits include many bioactive 3,4-seco-triterpenoids. *A. sessiliflorus* fruits were extracted in aqueous EtOH and fractionated into EtOAc, *n*-BuOH, and H_2_O fractions. Repeated column chromatographies for the organic fractions led to the isolation of 3,4-seco-triterpenoid glycosides, including new compounds. Ultra-high-performance liquid chromatography (UPLC) mass spectrometry (MS) systems were used for quantitation and quantification. BV2 and RAW264.7 cells were induced by LPS, and the levels of pro-inflammatory cytokines and mediators and their underlying mechanisms were measured by ELISA and Western blotting. NMR, IR, and HR-MS analyses revealed the chemical structures of the nine noble 3,4-seco-triterpenoid glycosides, acanthosessilioside G–O, and two known ones. The amounts of the compounds were 0.01–2.806 mg/g, respectively. Acanthosessilioside K, L, and M were the most effective in inhibiting NO, PGE_2_, TNF-*α*, IL-1*β*, and IL-6 production and reducing iNOS and COX-2 expression. In addition, it had inhibitory effects on the LPS-induced p38 and ERK MAPK phosphorylation in both BV2 and RAW264.7 cells. Nine noble 3,4-seco-triterpenoid glycosides were isolated from *A. sessiliflorus* fruits, and acanthosessilioside K, L, and M showed high anti-inflammatory and anti-neuroinflammatory effects.

## 1. Introduction

Oxidative stress in the human body is caused by excess reactive oxygen or nitrogen species (ROS or RNS), including superoxide (O_2_^−^), hydrogen peroxide (H_2_O_2_), and nitric oxide (NO), and it triggers certain types of apoptotic cell death, such as neuronal cell death and macrophage immune injury. If ROS or RNS are not appropriately removed, oxidative stress can cause the lipid peroxidation of the cellular membrane, leading to inordinate cell death [1].

Recently, the number of patients with degenerative brain disease has increased due to the aging society [2]. Therefore, the investigation of the cause and drug development for degenerative brain disease is rapidly progressing. The microglia in the central nerve system (CNS) plays an important role in the immunity, degeneration, and inflammation of the CNS, so it can prevent degenerative brain diseases by regulating the inflammatory response that occurs in microglials [3]. If the microglia overwhelmed by stimulation such as lipopolysaccharide (LPS), interferon-gamma (IFN-α), or tumor necrosis factor-alpha (TNF-α), NO, excessive cytokines, and ROS are secreted, causing neurotoxicity and destroying brain tissue, causing degenerative brain diseases such as Alzheimer’s, Parkinson’s disease, and Croyfeldt–Jakob disease [4,5,6]. Therefore, to prevent brain damage caused by ROS, RNS (NO), and excessive cytokines, it is useful to take anti-inflammatory and anti-neuroinflammatory supplements. However, synthetic anti-inflammatory agents can be toxic and carcinogenic and can interfere with the metabolic and respiratory activities of cells. Therefore, there is a need to develop alternative natural anti-inflammatory drugs without side effects.

The *Acanthopanax* genus is the deciduous broadleaf shrub in the family Araliaceae. *Acanthopanax* species are native to eastern Asia, from southeast Siberia and Japan to the Philippines and Vietnam [7]. *Acanthopanax* species are used commonly in traditional oriental medicine to treat cerebrovascular diseases, tumors, rheumatoid arthritis, diabetes, and hypertension [8]. *A. sessiliflorus* (Rupr. Et Maxim) is a deciduous broadleaf shrub that grows from 3 to 4 m in length, with sharp thorns on its branches. The barks are gray and the leaves are palmate leaves with five small leaves and have long petioles. Flowers are yellow-green and, in spring, hang in an umbel at the end of a long peduncle, and each flower has five petals. Fruits are ball-shaped, 6–7 mm in diameter, and ripen black around September [8,9]. Previous research has reported that triterpenoids and lignans are thought to be the active constituents in this plant [10,11,12]. Our previous study reported the isolation and identification of 3,4-seco-lupane triterpenes and its glycosides from an EtOAc fraction of *A. sessiliflorus* fruits, and their cytotoxicity against six human cancer cell lines and their ability to inhibit LPS-induced NO production in RAW 264.7 macrophages were shown [8]. Therefore, it is predictable that *A. sessiliflorus* fruits contain more various bioactive triterpene glycosides. Furthermore, recent reports have stated that an ethanol extract of *A. sessiliflorus* has antioxidant, antibacterial [13], antihypertensive [14], and anti-inflammatory [15] effects by these components.

In this study, we isolated and identified the 3,4-seco triterpenoid glycosides in *A. sessiliflorus* fruits and the isolated compounds were evaluated for their anti-inflammatory and anti-neuroinflammatory activities in RAW 264.7 macrophage and BV2 microglia.

## 2. Materials and Methods

### 2.1. Plant Materials

*A. sessiliflorus* fruits were harvested from the Pyeongchang county, Gangwon province, Korea (latitude 37°6′ N, longitude 128°3′ E). A voucher specimen (MPS006299) was deposited at the Herbarium of the Department of Herbal Crop Research, National Institute of Horticultural and Herbal Science, Rural Development Administration, Eumseong, Korea.

### 2.2. General Experimental Procedures

The materials and equipment we used for the isolation and structure determination of constituents are described in a previous study [16]. Tissue culture reagents, such as Roswell Park Memorial Institute 1640 (RPMI1640) and fetal bovine serum, were purchased from Gibco BRL Co. (Grand Island, NY, USA). All chemicals were obtained from Sigma-Aldrich Chemical Co. (St. Louis, MO, USA). Primary antibodies, including anti-iNOS, anti-COX-2, and anti-β-actin, were purchased from Santa Cruz Biotechnology (Santa Cruz, CA, USA); anti-p-JNK, anti-JNK, anti-p-ERK, anti-ERK, anti-p-p38, and anti-p38 were purchased from Cell Signaling Technology (Danvers, MA, USA); anti-rabbit and anti-mouse secondary antibodies were purchased from Millipore (Billerica, MA, USA). Enzyme-linked immunosorbent assay (ELISA) kits for PGE_2_, IL-6, and TNF-α assessments were purchased from R&D Systems, Inc. (Minneapolis, MN, USA). The compound structure was determined by analyzing various spectroscopic data, including mass spectroscopy and nuclear magnetic resonance, which were consistent with those previously reported [17].

### 2.3. Extraction and Isolation

The dried and powdered fruits of *A. sessiliflorus* (5 kg) were extracted with 70% aqueous ethanol (MeOH, 10 L × 2) for 24 h, which yielded a concentrated extract (ASFE, 1211 g). The concentrated extract was suspended in water (2 L) and successively extracted with EtOAc (2 L × 2) and *n*-BuOH (1.6 L × 2) to yield concentrated extracts of the EtOAc (AFE), *n*-BuOH (AFB, 88 g), and H_2_O (AFH) fractions. The concentrated *n*-BuOH fraction (AFB, 80 g) was subjected to silica gel CC (15 × 25 cm) using a gradient of CHCl_3_-MeOH-H_2_O (15:3:1→12:3:1→7:3:1→65:35:10) to yield ten fractions (B1 to 10). Fraction B1 (5 g) was subjected to silica gel CC (4 × 18 cm, CHCl_3_-MeOH-H_2_O (12:3:1→9:3:1)) to yield twenty-two subfractions (FAB1-1 to 22). Subfractions (FAB1-17, 50 mg) were separated by CC (RP-10 (2 × 8 cm), MeOH-H_2_O (2:1)) to yield Compound **1** (21 mg). Subfractions (FAB1-22, 80 mg) were separated by CC (RP-10 (2 × 10 cm), MeOH-H_2_O (2:1)) to yield Compounds **2** (8 mg) and **4** (17 mg). Fraction B4 (11 g) was subjected to silica gel CC (5 × 15 cm, CHCl_3_-MeOH-H_2_O (9:3:1→7:3:1)) to yield twenty subfractions (FAB4-1 to 20). Subfractions (FAB4-1, 180 mg) were fractionated using silica gel CC (3.5 × 20 cm, CHCl_3_-MeOH-H_2_O (7:3:1)) and yielded seven subfractions (FAB4-1-1 to 7). Subfractions (FAB4-1-7, 40 mg) were purified using CC (RP-18 (2 × 6 cm), MeOH-H_2_O (3:1)) and yielded Compound **10** (14 mg). Subfractions (FAB4-12-24, 21 mg) were separated by CC (RP-10 (1 × 10 cm), MeOH-H_2_O (1:1)) to yield Compound **5** (12 mg). Subfractions (FAB4-12-26, 105 mg) were separated by CC [RP-10 (3 × 10 cm), MeOH-H_2_O (2:1)] to yield Compounds **8** (25 mg) and **9** (13 mg). Subfractions (FAB4-12-27, 33 mg) were separated by CC (RP-10 (1.5 × 8 cm), MeOH-H_2_O (1:1)) to yield Compound **6** (8.5 mg). Subfractions (FAB4-12-29, 25 mg) were separated by CC (RP-10 (1.5 × 8 cm), MeOH-H_2_O (1.5:1)) to yield Compound **3** (12.3 mg). Subfractions (FAB4-12-29, 70 mg) were chromatographed (RP-18 (3.5 × 6.5 cm), MeOH-H_2_O (2:1)) to yield Compound **7** (28 mg). Fraction B7 (3.3 g) was fractionated using silica gel CC (4 × 12 cm, CHCl_3_-MeOH-H_2_O (7:3:1)) and yielded nine subfractions (FAB7-1 to 9). Subfractions (FAB7-5, 330 mg) were purified using CC (RP-18 (3.5 × 10 cm), MeOH-H_2_O (3:1)) and yielded ten subfractions (FAB7-5-1 to 10). Purification of Subfractions FAB7-5-6 (80 mg) using CC (RP-18 (3 × 6 cm), MeOH-H_2_O (2:1)) yielded Compound **11** (25 mg).

Acanthosessilioside G [22*α*-hydroxy-3,4-seco-lupa-4(23),20(30)-diene-3,28-dioic acid 28-*O*-*β*-d-glucopyranosyl-(1→2)-*β*-d-glucopyranose] (1): White amorphous powder; IR (CaF_2_ window) 3360, 1735, 1650 cm^−1^; negative ESI-QTOF/MS m/z 809.4448 [M−H]^−^ (calculated for C_42_H_65_O_15_, 809.4323); ^1^H-NMR (400 MHz, pyridine-*d*_5_, δ_H_) and ^13^C-NMR (100 MHz, pyridine-*d*_5_, δ_C_) refer to Table 1 and Table 2.

Acanthosessilioside H [22*α*-hydroxy-3,4-seco-lupa-4(23),20(30)-diene-3,28-dioic acid 28-[*α*-rhamnopyranosyl-(1→4)-*β*-glucopyranosyl-(1→6)-*β*-glucopyranose]] (2): White amorphous powder; IR (CaF_2_ window) 3356, 1715, 1609 cm^−1^; negative ESI-QTOF/MS m/z 955.5059 [M−H]^−^ (calculated for C_48_H_75_O_19_, 955.4902); ^1^H-NMR (400 MHz, pyridine-*d*_5_, δ_H_) and ^13^C-NMR (100 MHz, pyridine-*d*_5_, δ_C_) refer to Table 1 and Table 2.

Acanthosessilioside I [22*α*-hydroxy-3,4-seco-lupa-4(23),20(30)-diene-3,28-dioic acid 3-methyl ester 28-[*α*-rhamnopyranosyl-(1→4)-*β*-glucopyranosyl-(1→6)-*β*-glucopyranose]] (3): White amorphous powder; IR (CaF_2_ window) 3354, 1751, 1639 cm^−1^; negative ESI-QTOF/MS m/z 969.5034 [M−H]^−^ (calculated for C_49_H_77_O_19_, 969.5059); ^1^H-NMR (400 MHz, pyridine-*d*_5_, δ_H_) and ^13^C-NMR (100 MHz, pyridine-*d*_5_, δ_C_) refer to Table 1 and Table 2.

Sessiloside (4): White amorphous powder; IR (CaF_2_ window) 3355, 1715, 1640 cm^−1^; negative ESI-QTOF/MS m/z 939.5123 [M−H]^−^; ^1^H-NMR (400 MHz, pyridine-*d*_5_, δ_H_) and ^13^C-NMR (100 MHz, pyridine-*d*_5_, δ_C_) data were consistent with those in [18].

Acanthosessilioside J [3,4-seco-lupa-4(23),20(30)-diene-3,28-dioic acid 3-methyl ester 28-[*α*-rhamnopyranosyl-(1→4)-*β*-glucopyranosyl-(1→6)-*β*-glucopyranose]] (5): White amorphous powder; IR (CaF_2_ window) 3349, 1710, 1648 cm^−1^; negative ESI-QTOF/MS m/z 953.5128 [M−H]^−^ (calculated for C_49_H_77_O_18_, 953.5109); ^1^H-NMR (400 MHz, pyridine-*d*_5_, δ_H_) and ^13^C-NMR (100 MHz, pyridine-*d*_5_, δ_C_) refer to Table 1 and Table 2.

Inermoside (6): White amorphous powder; IR (CaF_2_ window) 3351, 1725, 1677 cm^−1^; negative FAB-MS m/z 969 [M−H]^−^; ^1^H-NMR (400 MHz, pyridine-*d*_5_, δ_H_) and ^13^C-NMR (100 MHz, pyridine-*d*_5_, δ_C_) data were consistent with those in [19].

Acanthosessilioside K [(1*R*)-22*α*-propoxy-1*α*, 11*α*-dihydroxy-3,4-seco-lupa-4(23),20(30)-diene-3,28-dioic acid 3, 11-lactone 28-[*α*-rhamnopyranosyl-(1→4)-*β*-glucopyranosyl-(1→6)-*β*-glucopyranose]] (7): White amorphous powder; IR (CaF_2_ window) 3360, 1714, 1627 cm^−1^; negative ESI-QTOF/MS m/z 1011.5530 [M−H]^−^ (calculated for C_51_H_79_O_20_, 1011.5165); ^1^H-NMR (400 MHz, pyridine-*d*_5_, δ_H_) and ^13^C-NMR (100 MHz, pyridine-*d*_5_, δ_C_) refer to Table 1 and Table 2.

Acanthosessilioside L [(1*R*)-1,4-epoxy-3,4-seco-lup-20(30)-ene-3,28-dioic acid 3-methyl ester 28-[*α*-rhamnopyranosyl-(1→4)-*β*-glucopyranosyl-(1→6)-*β*-glucopyranose]] (8): White amorphous powder; IR (CaF_2_ window) 3360, 1720, 1615 cm^−1^; negative ESI-QTOF/MS m/z 969.5034 [M−H]^−^ (calculated for C_49_H_77_O_19_, 969.5059); ^1^H-NMR (400 MHz, pyridine-*d*_5_, δ_H_) and ^13^C-NMR (100 MHz, pyridine-*d*_5_, δ_C_) refer to Table 1 and Table 2.

Acanthosessilioside M [(1*R*)-1,4-epoxy-11*α*-propoxy-3,4-seco-lup-20(30)-ene-3,28-dioic acid 3-methyl ester 28-[*α*-rhamnopyranosyl-(1→4)-*β*-glucopyranosyl-(1→6)-*β*-glucopyranose]] (9): White amorphous powder; IR (CaF_2_ window) 3348, 1736, 1648 cm^−1^; negative ESI-QTOF/MS m/z 1027.5483 [M−H]^−^ (calculated for C_52_H_83_O_20_, 1027.5478); ^1^H-NMR (400 MHz, pyridine-*d*_5_, δ_H_) and ^13^C-NMR (100 MHz, pyridine-*d*_5_, δ_C_) refer to Table 1 and Table 2.

Acanthosessilioside N [(1*R*)-1,4-epoxy-11*α*, 22*α*-dihydroxy-3,4-seco-lup-20(30)-ene-3,28-dioic acid 3-methyl ester 28-[*α*-rhamnopyranosyl-(1→4)-*β*-glucopyranosyl-(1→6)-*β*-glucopyranose]] (10): White amorphous powder; IR (CaF_2_ window) 3351, 1715, 1640 cm^−1^; negative ESI-QTOF/MS m/z 1001.4948 [M−H]^−^ (calculated for C_49_H_77_O_21_, 1001.4957); ^1^H-NMR (400 MHz, pyridine-*d*_5_, δ_H_) and ^13^C-NMR (100 MHz, pyridine-*d*_5_, δ_C_) refer to Table 1 and Table 2.

Acanthosessilioside O [1, 11*α*, 22*α*-trihydroxy-3,4-seco-lupa-4(23),20(30)-diene-3,28-dioic acid 3-methyl ester 28-[*α*-rhamnopyranosyl-(1→4)-*β*-glucopyranosyl-(1→6)-*β*-glucopyranose]] (11): White amorphous powder; IR (CaF_2_ window) 3356, 1750, 1615 cm^−1^; negative ESI-QTOF/MS m/z 1001.4948 [M−H]^−^ (calculated for C_49_H_77_O_21_, 1001.1957); ^1^H-NMR (400 MHz, pyridine-*d*_5_, δ_H_) and ^13^C-NMR (100 MHz, pyridine-*d*_5_, δ_C_) refer to Table 1 and Table 2.

### 2.4. UPLC-QTOF/MS and UPLC-MS/MS Analyses

UPLC was performed using a Waters ACUITY I-CLASS UPLC (Waters Corp., Milford, MA, USA) with a Thermo Hypersil GOLD column (2.1 mm × 100 mm; 1.9 μm). The mobile phases were composed with Solvent A (water and 0.1% formic acid (v/v)) and Solvent B (acetonitrile and 0.1% formic acid (v/v)). The flow rate was 450 μL/min and the injection volume was 2 μL. The elution conditions were as follows: 0–4 min, B 10–30%; 4–15 min, B 30–60%; 15–16 min, B 60–100%; 16–18 min, B 100–10. The column oven and sample tray were maintained at 40 ℃ and 4 ℃, respectively. MS analysis was performed using a Waters Xevo G2-S QTOF MS (Waters Corp., Milford, MA, USA) operating in negative ion mode. Accurate mass measurements were obtained by an automated calibration delivery system that contained an internal reference (Leucine-enkephalin, m/z 554.262 (ESI-)). The operating parameters were set by modifying those from the preceding research [20]. UPLC with tandem mass spectrometry (MS/MS) was carried out for the quantitative analysis of compounds in the *A. sessiliflorus* fruits. Mass spectrometric detection was carried out using 3200 QTRAP mass spectrometer (AB SCIEX, Framingham, MA, USA) in multiple reaction monitoring (MRM) mode. Precursor ions of the compounds were selected. They and product ions were generated by applying collision energies to selected precursor ions, and product ions were used for quantification. The optimal operating parameters were set as shown in Table 3.

### 2.5. Cell Culture and Viability Assays

BV2 and RAW264.7 cells were incubated at 5 × 10^5^ cells/mL in RPMI1640 containing 1% antibiotic (Penicillin-Streptomycin) and 10% heat-inactivated FBS at 37 °C in a humidified 5% CO_2_ and a 95% air atmosphere. These cells were incubated according to a previously described method [21,22].

### 2.6. Determination of Nitrite

As an indicator of nitrite oxide (NO) production in cells, the production of nitrite, a stable end-product of NO oxidation, was measured. Briefly, the concentration of nitrite in the conditioned media was determined by a method based on the Griess reaction [23]. The details of the assay have been described previously [21].

### 2.7. PGE_2_ Assay

The concentration of PGE_2_ in each sample was measured with the use of a commercially available kit from R&D Systems, Inc (Minneapolis, MN, USA), according to a method described previously [21]. Briefly, BV2 and RAW264.7 cells treated with compounds were cultured in 48-well plates, followed by a pre-incubation with different concentrations of compounds for 3 h. Subsequently, stimulation was performed for 24 h with LPS stimulation (1 μg/mL). The resulting cell culture supernatants were collected and centrifuged at 13,000× *g* for 2 min to remove particulate matter. At the end of the procedure, the samples were added to a 96-well plate pre-coated with polyclonal antibodies specific to PGE_2_. Enzyme-linked polyclonal antibodies were added to the wells and allowed to react for 20 h, followed by a final washing step to remove any unbound antibody-enzyme reagent. A substrate solution was added, after which the intensity of the color produced was measured at 450 nm (a correction wavelength set at 540 or 570 nm), which was proportional to the amount of PGE_2_ present.

### 2.8. Assays for IL-1β, IL-6, and TNF-α

The culture medium was collected to determine the levels of IL-β, IL-6, and TNF-α present in each sample using ELISA kits tailored to each collection process (R&D Systems, Inc.), as per the manufacturer’s instructions. Briefly, BV2 and RAW264.7 cells were seeded in 48-well culture plates at a density of 5 × 10^5^ cells/well. After incubation, the supernatant was collected and used in the cytokine ELISA kits for measuring the concentrations of IL-1β, IL-6, and TNF-α.

### 2.9. Western Blotting Analysis

The pelleted BV2 and RAW264.7 cells were washed with PBS and then lysed in RIPA buffer. Equal amounts of proteins quantified by Protein Assay Dye Reagent Concentrate obtained from Bio-Rad Laboratories (#5000006; Hercules, CA, USA), mixed in the sample loading buffer and separated by SDS-PAGE. The separated proteins were transferred to a nitrocellulose membrane. Non-specific binding to the membrane was blocked by incubation in a solution of skimmed milk. The membrane was incubated with primary antibodies at 4 °C overnight and then reacted with a horseradish peroxidase-conjugated secondary antibody from Millipore.

### 2.10. Statistical Analysis

Data are presented as the mean ± standard deviation of three independent experiments. A one-way analysis of variance, followed by Dunnett’s comparison tests, was used to compare the two groups. Statistical analyses were performed using GraphPad Prism software, Version 5.01 (GraphPad Software Inc., San Diego, CA, USA).

## 3. Results and Discussion

### 3.1. Chemical Structure Elucidation of Compounds **1**–**11**

**Figure 1:**
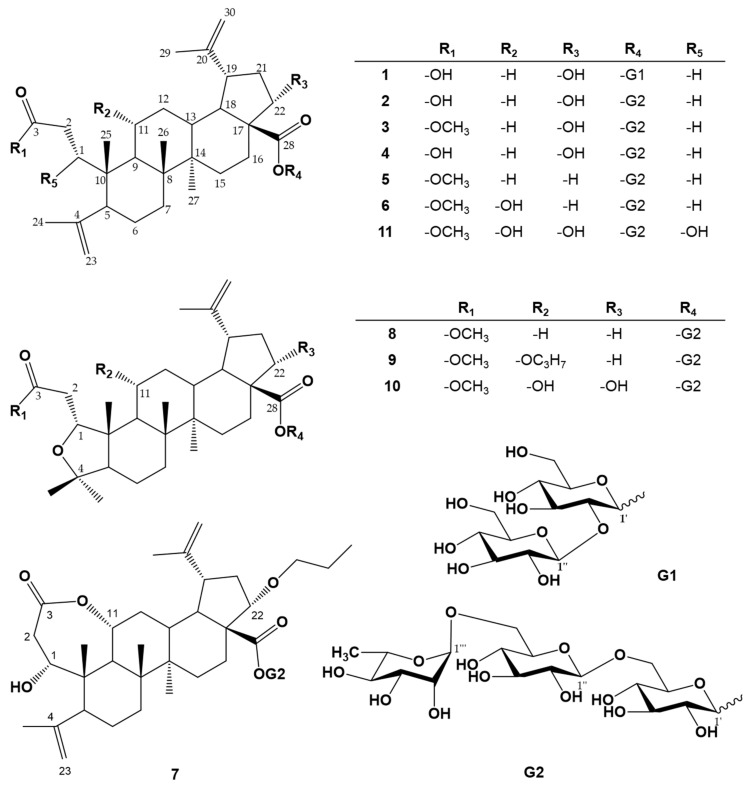
Chemical structures of compounds **1**–**11** from the fruits of *Acanthopanax sessiliflorus.*

A 70% ethanolic extract of dried *A. sessiliflorus* fruits was suspended in H_2_O and extracted successively with EtOAc and *n*-BuOH. The EtOAc- and *n*-BuOH-soluble fractions were concentrated under reduced pressure to produce a residue that was subjected to multiple chromatographic steps, using Diaion HP-20, Sephadex LH-20, silica gel, and reversed-phase C18 silica gel, yielding compounds **1**–**11**. Comparing the 1D- and 2D-NMR and QTOF/MS data with reported values allowed us to identify known compounds sessiloside (**4**) [18] and inermoside (**6**) [19]. The other nine noble compounds are newly reported here (Figure 1).

The infrared spectrum (cm^−1^) of compounds **1**–**3**, **5**, and **7**–**11** suggested the presence of a double bond (1665, 1640), a carbonyl (1746, 1710), and a hydroxyl (3462, 3342). Compound **1** was obtained as a white, amorphous powder. The molecular formula was determined to be C_42_H_66_O_15_ from the pseudomolecular ion peak [M−H]^−^ at m/z 809.4448 (calculated for C_42_H_65_O_15_, 809.4323) in the negative HR-QTOF/MS. In the ^1^H NMR spectrum (Table 1), the observation of signals for two allyl methyl protons (chemical shift, coupling pattern, *J* in Hz, proton number; δ_H_ 1.71, H-24; δ_H_ 2.13, H-29) located at sp^2^ carbons and four olefinic methine protons (δ_H_ 5.12, d, 2.0, H-23a, 4.83, 2.0, H-23b; δ_H_ 4.90, overlap, H-30a, 4.79, overlap, H-30b), of which the chemical shifts and small coupling constants (or broad singlet) were typical of two exomethylene units, confirmed the presence of two isopropenyl moieties. In addition, signals for three tertiary methyl protons (δ_H_ 1.21, s, H-26; δ_H_ 1.12, s, H-25, δ_H_ 0.80, s, H-27) and an oxygenated methine proton (δ_H_ 4.80, overlap, H-22) were observed. The ^1^H NMR signals of the two sugars included two hemiacetals (δ_H_ 6.37, d, 8.0, H-1′; δ_H_ 5.35, d, 8.0, H-1′′), eight oxygenated methines (δ_H_ 4.00, overlap, H-5″; δ_H_ 4.00, overlap, H-5′; δ_H_ 4.07, overlap, H-2″; δ_H_ 4.27, overlap, H-3″; δ_H_ 4.30, overlap, H-4″; δ_H_ 4.30, overlap, H-4′; δ_H_ 4.30, overlap, H-2′; δ_H_ 4.37, overlap, H-3′), and two oxygenated methylenes (δ_H_ 4.34, overlap, H-6″b, 4.42, 1H, br. d, 12.0, H-6″a; δ_H_ 4.46, overlap, H-6′b, 4.58, 1H, br. d, 12.0, H-6′a). The ^13^C NMR spectrum of **1** (Table 2) supported by DEPT experiments indicated the presence of 30 carbons including two carbonyl carbons (δ_C_ 173.6, C-3; δ_C_ 179.0, C-28), two olefinic quaternary carbons (δ_C_ 152.1, C-20; δ_C_ 148.1, C-4), two exomethylene carbons (δ_C_ 114.4, C-23; δ_C_ 110.9, C-30), an oxygenated methine carbon (δ_C_ 76.0, C-22), five methyl carbons (δ_C_ 23.6, C-24; δ_C_ 20.5, C-25; δ_C_ 19.5, C-29; δ_C_ 16.5, C-26; δ_C_ 15.0, C-27), and 18 other carbon signals. The chemical shifts in the ^13^C NMR signals due to two hemiacetals (δ_C_ 94.3, C-1″; δ_C_ 107.1, C-1′), eight oxygenated methines (δ_C_ 71.1, C-4′; δ_C_ 71.8, C-4′′; δ_C_ 76.8, C-2″; δ_C_ 78.5, C-3″; δ_C_ 78.6, C-3′; δ_C_ 79.2, C-5′; δ_C_ 79.7, C-5″; δ_C_ 83.6, C-2′), and two oxygenated methylenes (δ_C_ 62.5, C-6′; δ_C_ 62.4, C-6″) and the coupling constant of the anomer proton signal (*J* = 8.0 Hz) revealed that the two sugars were *β*-glucopyranosyl-(1→2)-*β*-glucopyranose. The oxygenated methine carbon signal (C-2′) was down-shifted at δ_C_ 83.6 owing to the glycosidation effect from their usual detection at δ_C_ 74.5 in *β*-d-glucopyranose [16]. In addition, the anomer carbon signal (C-1′) was up-shifted at δ_C_ 94.3 owing to the esterification effect from their usual detection at δ_C_ 105.0 in *β*-d-glucopyranose [8]. Several cross-peaks in the ^1^H−^1^H COSY spectrum confirmed a key connection among the proton signals. The HMBC spectrum showed crucial long-range correlations between H-23a, H-23b/C-5; H-2a, H-2b/C-3; and H-1/C-3. Therefore, **1** was assigned as a 3,4-secolupane-type triterpenoid. In addition, two anomer proton signals (δ_H_ 6.33, H-1′; δ_H_ 4.94, H-1″) showed cross peaks with the oxygenated olefin quaternary carbon signal (δ_C_ 179.0, C-28) and the one oxygenated methine (δ_C_ 83.6, C-2′). On further analysis of the HSQC and DEPT 135 spectra of **1**, the assignments of the proton and carbon NMR signals (Table 1 and Table 2) were confirmed unambiguously. The coupling constant between H-22 and H-21 was 4.4 Hz, indicating that OH-22 is α-oriented. Therefore, the structure of compound **1** was determined as 22*α*-hydroxy-3,4-seco-lupa-4(23),20(30)-diene-3,28-dioic acid 28-*O*-*β*-d-glucopyranosyl-(1→2)-*β*-d-glucopyranose, which has not been previously reported. This compound was named acanthosessilioside G (Figure S1).

Compound **2** was obtained as a white, amorphous powder. The molecular formula was determined to be C_48_H_76_O_19_ from the pseudomolecular ion peak [M−H]^−^ at m/z 955.5059 (calculated for C_48_H_75_O_19_, 955.4902) in the negative HR-QTOF/MS. The ^1^H NMR and ^13^C NMR of compound **2** were similar to those of compound 1, except for signals indicating an additional monosaccharide moiety. The ^1^H NMR signals of the monosaccharide included a hemiacetal (δ_H_ 5.83, br. s, H-1‴), four oxygenated methines (δ_H_ 3.93, dd, 8.4, 8.4, H-4‴; δ_H_ 4.53, dd, 8.4, 3.2, H-3‴; δ_H_ 4.66, overlap, H-2‴; δ_H_ 4.93, overlap, H-5‴), and one methyl (δ_H_ 1.71, d, 6.0, H-6‴). The chemical shifts in the ^13^C NMR signals due to a hemiacetal (δ_C_ 103.2, C-1‴), four oxygenated methines (δ_C_ 70.7, C-5″; δ_C_ 72.9, C-2‴; δ_C_ 73.1, C-3‴; δ_C_ 74.4, C-4‴), and one methyl (δ_C_ 18.9, C-6‴) and the coupling constant of the anomer proton signal (br. s) revealed that the monosaccharide was a *α*-rhamnopyranose. In the gHMBC spectrum, three anomer proton signals (δ_H_ 6.38, H-1′; δ_H_ 5.70, H-1‴; δ_H_ 4.94, H-1″) showed cross peaks with the ester carbon signal (δ_C_ 177.0, C-28), the oxygenated methylene carbon signal (δ_C_ 70.0, C-6′), and the oxygenated methine carbon signal (δ_C_ 76.9, C-4′′), respectively, which revealed that the three sugars were *α*-rhamnopyranosyl-(1→4)-*β*-glucopyranosyl-(1→6)-*β*-glucopyranose. Consequently, the structure of compound **2** was determined as 22*α*-hydroxy-3,4-seco-lupa-4(23),20(30)-diene-3,28-dioic acid 28-[*α*-rhamnopyranosyl-(1→4)-*β*-glucopyranosyl-(1→6)-*β*-glucopyranose], which has not been previously reported. This compound was named acanthosessilioside H (Figure S2).

Compound **3** was obtained as a white, amorphous powder. The molecular formula was determined to be C_49_H_78_O_19_ from the pseudomolecular ion peak [M−H]^−^ at m/z 969.5034 (calculated for C_49_H_77_O_19_, 969.5059) in the negative HR-QTOF/MS. The ^1^H NMR and ^13^C NMR of compound **3** were similar to those of compound **2**, except for signals indicating an additional one methoxy group signal (δ_H_ 3.63, s; δ_C_ 51.3, 3-OCH_3_). In the gHMBC spectrum, the methoxy proton signal (δ_H_ 3.63) displayed cross peaks with the ester carbon signal (δ_C_ 174.3, C-3), suggesting that the methoxy groups were at the C-3 positions. When compared with a previously reported compound, acanthosessiligenins I [5], the structure of compound **3** was determined as 22*α*-hydroxy-3,4-seco-lupa-4(23),20(30)-diene-3,28-dioic acid 3-methyl ester 28-[*α*-rhamnopyranosyl-(1→4)-*β*-glucopyranosyl-(1→6)-*β*-glucopyranose], which has not been previously reported. This compound was named acanthosessilioside I (Figure S3).

Compound **5** was obtained as a white, amorphous powder. The molecular formula was determined to be C_49_H_78_O_18_ from the pseudomolecular ion peak [M−H]^−^ at m/z 953.5128 (calculated for C_49_H_77_O_18_, 953.5109) in the negative HR-QTOF/MS. The ^1^H NMR and ^13^C NMR of Compound **5** were similar to those of Compound **3**, except for signals indicating an exceptional one hydroxy group signal (δ_H_ 1.73, overlap, H-22a, 1.36, overlap, H-22b; δ_C_ 36.8, C-22). When compared with a previously reported compound, acanthosessiliosides A [8], the structure of compound **5** was determined as 3,4-seco-lupa-4(23),20(30)-diene-3,28-dioic acid 3-methyl ester 28-[*α*-rhamnopyranosyl-(1→4)-*β*-glucopyranosyl-(1→6)-*β*-glucopyranose], which has not been previously reported. This compound was named acanthosessilioside J (Figure S4).

Compound **7** was obtained as a white, amorphous powder. The molecular formula was determined to be C_51_H_80_O_20_ from the pseudomolecular ion peak [M−H]^−^ at m/z 1011.5530 (calculated for C_51_H_79_O_20_, 1011.5165) in the negative HR-QTOF/MS. Its ^1^H NMR and ^13^C NMR spectra (Table 1 and Table 2) were similar to those of 22*α*-hydroxychiisanoside [12], except for signals indicating an additional propoxy group signal (δ_H_ 4.80, overlap, H-22; δ_C_ 67.7, C-11; δ_H_ 4.42, overlap, H-1⁗; δ_C_ 69.5, C-1⁗; δ_H_ 2.05, overlap, H-2⁗; δ_C_ 19.4, C-2⁗; δ_H_ 0.79, overlap, H-3⁗; δ_C_ 13.7, C-3⁗). The sugar moieties were similar to those of the sugars in compound **5**. In the gHMBC spectrum, the oxygenated methine proton signal (δ_H_ 4.80, H-22) displayed a cross peak with one ester carbon signals (δ_C_ 174.0, C-28) and one oxygenated methylene carbon signal (δ_C_ 69.5, C-1⁗), suggesting that the oxygenated methine group was at the C-22 positions and attached with a propoxy group. Consequently, the structure of compound **7** was determined as (1*R*)-22*α*-propoxy-1*α*, 11*α*-dihydroxy-3,4-seco-lupa-4(23),20(30)-diene-3,28-dioic acid 3,11-lactone 28-[*α*-rhamnopyranosyl-(1→4)-*β*-glucopyranosyl-(1→6)-*β*-glucopyranose], which has not been previously reported. This compound was named acanthosessilioside K (Figure S5).

Compound **8** was obtained as a white, amorphous powder. The molecular formula was determined to be C_49_H_78_O_19_ from the pseudomolecular ion peak [M−H]^−^ at m/z 969.5034 (calculated for C_49_H_77_O_19_, 969.5059) in the negative HR-QTOF/MS. The ^1^H and ^13^C NMR spectra (Table 1 and Table 2) exhibited signals for five tertiary methyl groups (δ_H_ 1.10, 1.21, 1.24, 1.32, 1.37), an allyl methyl group (δ_H_ 1.93), an exomethlyene group (δ_H_ 4.91/4.66) due to an isopropenyl moiety, one oxygenated methine group (δ_H_ 4.86, dd, 12.0, 2.4, H-1; δ_C_ 86.6, C-1), a methoxy group (δ_H_ 3.60, s; δ_C_ 51.1, 3-OCH_3_), and two carbonyl groups (δ_C_ 173.4, C-3; δ_C_ 174.7, C-28). When compared with a previously reported compound, acanthosessiligenin II [8], 8 was found to lack a hydroxyl group at the C-22 position but to possess an additional *α*-rhamnopyranosyl-(1→4)-*β*-glucopyranosyl-(1→6)-*β*-glucopyranose group. In the gHMBC spectrum, three anomer proton signals (δ_H_ 6.37, H-1′; δ_H_ 5.85, H-1‴; δ_H_ 4.92, H-1″) showed cross peaks with the ester carbon signal (δ_C_ 174.7, C-28), the oxygenated methylene carbon signal (δ_C_ 69.5, C-6′), and the oxygenated methine carbon signal (δ_C_ 76.3, C-4′′), respectively. In addition, the long-range correlations between the methoxy protons signal (δ_H_ 3.60) and the carbonyl carbon signal at C-3 (δ_C_ 173.4) revealed that the methoxy group is affixed to C-3. Consequently, the structure of compound **8** was determined as (1*R*)-1,4-epoxy-3,4-seco-lup-20(30)-ene-3,28-dioic acid 3-methyl ester 28-[*α*-rhamnopyranosyl-(1→4)-*β*-glucopyranosyl-(1→6)-*β*-glucopyranose], which has not been previously reported. This compound was named acanthosessilioside L (Figure S6).

Compound **9** was obtained as a white, amorphous powder. The molecular formula was determined to be C_52_H_84_O_20_ from the pseudomolecular ion peak [M−H]^−^ at m/z 1027.5483 (calculated for C_52_H_83_O_20_, 1027.5478) in the negative HR-QTOF/MS. The ^1^H NMR and ^13^C NMR of compound **9** were similar to those of compound **8**, except for signals indicating an additional one propoxy group signals (δ_H_ 4.07, overlap, H-11; δ_C_ 67.7, C-11; δ_H_ 4.37, overlap, H-1⁗; δ_C_ 69.4, C-1⁗; δ_H_ 2.01, overlap, H-2⁗; δ_C_ 19.3, C-2⁗; δ_H_ 0.76, overlap, H-3⁗; δ_C_ 13.7, C-3⁗). In the gHMBC spectrum, one oxygenated methine proton signals (δ_H_ 4.07, H-11) displayed cross peaks with two methyl carbon signals (δ_C_ 18.8, C-25; δ_C_ 17.9, C-26) and one oxygenated methylene carbon signal (δ_C_ 69.4, C-1⁗), suggesting that one oxygenated methine groups were at the C-11 position and attached with the propoxy group. Consequently, the structure of compound **9** was determined as (1*R*)-1,4-epoxy-11*α*-propoxy-3,4-seco-lup-20(30)-ene-3,28-dioic acid 3-methyl ester 28-[*α*-rhamnopyranosyl-(1→4)-*β*-glucopyranosyl-(1→6)-*β*-glucopyranose], which has not been previously reported. This compound was named acanthosessilioside M (Figure S7).

Compound **10** was obtained as a white, amorphous powder. The molecular formula was determined to be C_49_H_78_O_21_ from the pseudomolecular ion peak [M−H]^−^ at m/z 1001.4948 (calculated for C_49_H_77_O_21_, 1001.4957) in the negative HR-QTOF/MS. The ^1^H NMR and ^13^C NMR of compound **10** were similar to those of compound **8**, except for signals indicating two additional hydroxy group signals (δ_H_ 4.07, overlap, H-11; δ_C_ 67.7, C-11; δ_H_ 4.80, overlap, H-22; δ_C_ 76.4, C-22). In the gHMBC spectrum, two oxygenated methine proton signals (δ_H_ 4.07, H-11; δ_H_ 4.80, H-22) displayed cross peaks with two methyl carbon signals (δ_C_ 20.4, C-25; δ_C_ 16.3, C-26) and one ester carbon signal (δ_C_ 174.7, C-28), suggesting that two oxygenated methine groups were at the C-11 and C-22 positions. Consequently, the structure of compound **10** was determined as (1*R*)-1,4-epoxy-11*α*, 22*α*-dihydroxy-3,4-seco-lup-20(30)-ene-3,28-dioic acid 3-methyl ester 28-[*α*-rhamnopyranosyl-(1→4)-*β*-glucopyranosyl-(1→6)-*β*-glucopyranose], which has not been previously reported. This compound was named acanthosessilioside N (Figure S8).

Compound **11** was obtained as a white, amorphous powder. The molecular formula was determined to be C_49_H_78_O_21_ from the pseudomolecular ion peak [M−H]^−^ at m/z 1001.4948 (calculated for C_49_H_77_O_21_, 1001.4957) in the negative HR-QTOF/MS. The ^1^H NMR and ^13^C NMR of compound **11** were similar to those of compound **7**, except for signals indicating an additional one hydroxy group signal (δ_H_ 4.86, overlap, H-1; δ_C_ 69.7, C-1) and one methoxy group signal (δ_H_ 3.60, s; δ_C_ 51.1, 3-OCH_3_). In the gHMBC spectrum, the oxygenated methine proton signal (δ_H_ 4.86, H-1) displayed cross peaks with one methyl carbon signal (δ_C_ 20.9, C-25) and one quaternary carbon signal (δ_C_ 45.9, C-10), suggesting that one oxygenated methine group was at the C-1 position. In addition, the long-range correlations between the methoxy protons signal (δ_H_ 3.60) and the carbonyl carbon signal at C-3 (δ_C_ 175.2) revealed that the methoxy group is affixed to C-3. Consequently, the structure of compound **11** was determined as 1,11*α*,22*α*-trihydroxy-3,4-seco-lupa-4(23),20(30)-diene-3,28-dioic acid 3-methyl ester 28-[*α*-rhamnopyranosyl-(1→4)-*β*-glucopyranosyl-(1→6)-*β*-glucopyranose], which has not been previously reported. This compound was named acanthosessilioside O (Figure S9).

### 3.2. Analyses of Compounds **1**–**11** in ASFE by UPLC-QTOF/MS and Tandem Mass Spectrometry (MS/MS)

**Figure 2:**
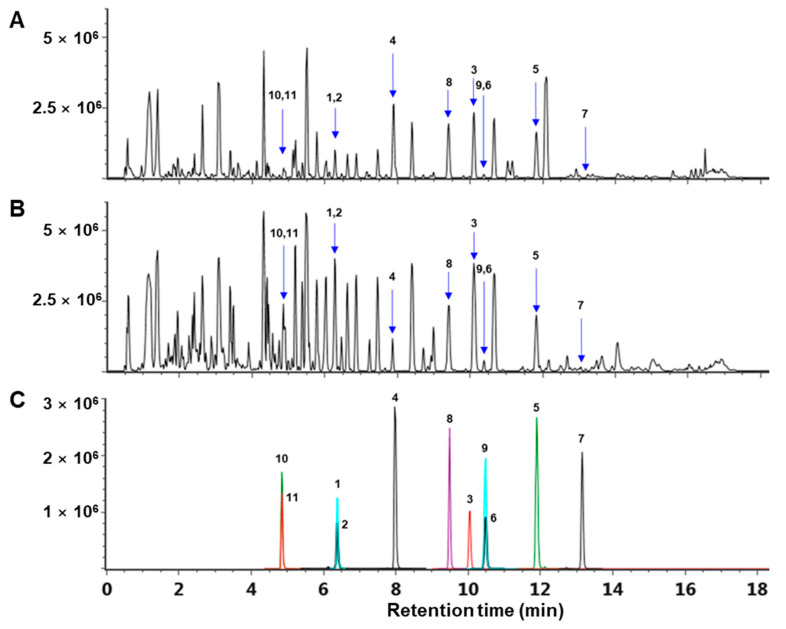
Representative negative ion mode mass chromatogram of (**A**) ASFEx, (**B**) AFB, and (**C**) isolated compounds (**1–11**) based on a UPLC(ESI)-QTOF-MS.

Compounds **1**–**11** were analyzed using ultra-high-performance liquid chromatography coupled with quadrupole time-of-flight mass spectrometry (UPLC-QTOF/MS). The extract of *A. sessiliflorus* fruits (ASFEx) was also subjected to UPLC-QTOF/MS. Four compounds, namely compound **1**–**3** and **5**, were determined in the extract but the intensities in the extract were too low. As a result of this, fractionation was carried out. A typical base peak intensity (BPI) chromatogram of the extract and the *n*-butanol fraction (AFB) are shown in Figure 2. After fractionation, eight compounds, **1**, **3**–**7**, **9**, and **10**, were detected in the UPLC chromatogram of the AFB. However, the intensities of compounds were low in the *n*-butanol fraction for quantitative analysis using UPLC-QTOF/MS. Hence, QTRAP^®^ tandem mass spectrometry (MS/MS) was used for quantifying compounds **1**–**11**. The precursor ions of noble compounds, acanthosessilioside G–O were selected under optimized multiple reaction monitoring (MRM) conditions, and collision energy was applied to the selected precursor ions. After that, the product ions with the highest intensities were selected for quantitative analysis (Table 4). The linear calibration curves were based on regression analysis of the values measured with various concentration of compounds **1**–**11**. The values of the calibration plots are shown in Table 5. The result of quantitative analysis was measured in the range of 0.01–2.806 mg/g.

### 3.3. Effects of Compounds **1**–**11** on Cell Viability and Nitrite Contents in BV2 and RAW264.7 Cells

**Figure 3:**
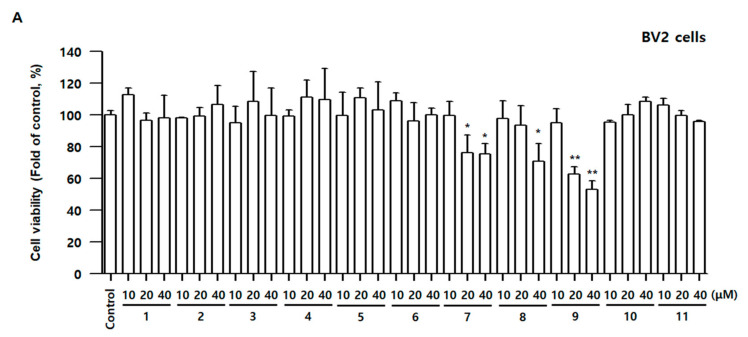

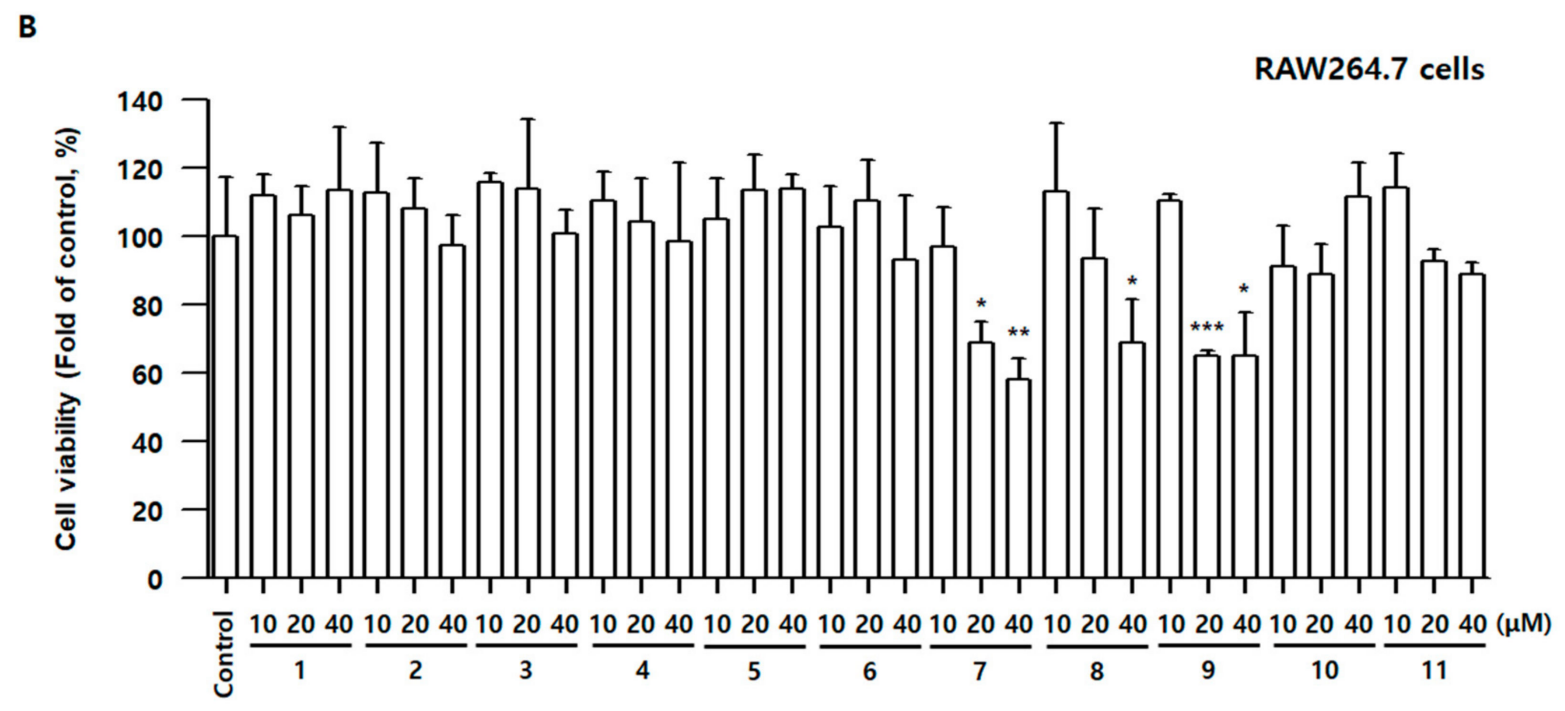
Effects of compounds **1**–**11** on cell viability in BV2 (**A**) and RAW264.7 (**B**) cells. The cells were incubated for 48 h with various concentrations of compounds **1**–**11**. Cell viability was determined using 3-(4,5-dimethylthiazol-2-yl)-2,5-diphenyltetrazolium bromide assay. Bars represent means ± standard deviation of three independent experiments. * *p* < 0.05, ** *p* < 0.01, *** *p* < 0.001 compared with control group.

To examine whether compounds **1**–**11** have cell toxicity, the 3-(4,5-dimethylthiazol-2-yl)-2,5-diphenyltetrazolium bromide assay was conducted in BV2 and RAW264.7 cells. The cells were incubated for 48 h with various concentrations of compounds **1**–**11**. The cell viability did not alter following the addition of 40 μM concentrations of compounds **1**–**6**, **10**, and **11** for 48 h; however, compound **7** at 20 μM, Compound **8** at 40 μM, and compound **9** at 20 μM demonstrated cell toxicity in BV2 and RAW264.7 cells. Therefore, adequate concentrations of compounds **1**–**11** in subsequent experiments were determined based on the results of Figure 3.

**Figure 4:**
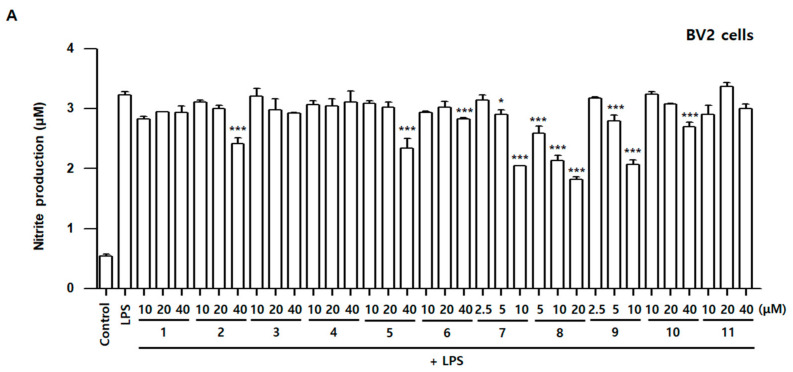

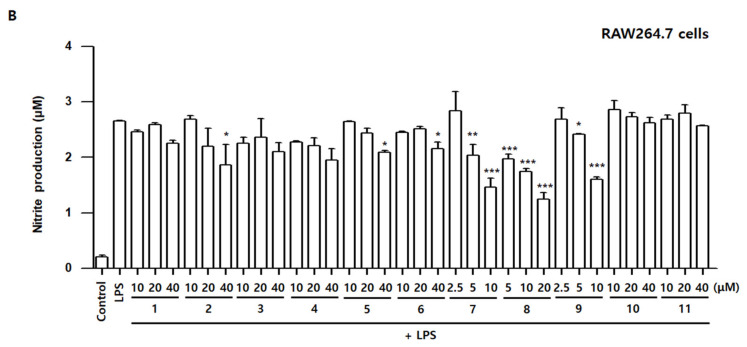
Effects of Compounds **1**–**11** on nitrite contents in LPS-stimulated BV2 (**A**) and RAW264.7 (**B**) cells. Cells were pretreated for 3 h with indicated concentrations of compounds **1**–**11** and stimulated for 24 h with LPS (1 μg/mL). Nitrite concentration was performed as described in Materials and Methods. Bars represent means ± standard deviation of three independent experiments. * *p* < 0.05, ** *p* < 0.01, *** *p* < 0.001 compared with the LPS-treated group.

To assess the anti-inflammatory and neuroinflammatory effects of compounds **1**–**11** in LPS-stimulated BV2 and RAW264.7 cells, nitrite concentration was measured using the Griess reagents. Over-production of iNOS-derived NO may promote the formation of RNS, aggravate the inflammatory response, and can even lead to neuronal cell death. LPS stimulates macrophage or/and microglia and advances the production of ROS [24]. Therefore, reducing ROS production in microglia and macrophage cells might be an effective strategy to protect against inflammatory damage. In other previous studies, LPS treatment significantly increased ROS production [25]. LPS-treated groups significantly increased nitrite concentration compared with the control group in both BV2 and RAW264.7 cells. In BV2 cells, the increased nitrite concentration was significantly inhibited by compounds **2**, **5**, **6**, **7**, **8**, **9**, and **10**. In RAW264.7 cells, the increased nitrite concentration was significantly inhibited by compounds **2**, **5**, **6**, **7**, **8**, and **9** (Figure 4). Among them, compounds **7**, 8, and 9 were the most effective in inhibiting nitrite production in both BV2 and RAW264.7 cells. These results showed that, among the eleven compounds, compounds **7**, **8**, and **9** are suitable for further experiments aimed at determining the biological mechanism.

### 3.4. Effects of Compounds **7**–**9** on Levels of Prostaglandin E2 (PGE_2_), Tumor Necrosis Factor (TNF)-α, Interleukin (IL)-1β, and Interleukin (IL)-6 in LPS-Stimulated BV2 and RAW264.7 Cells

**Figure 5:**
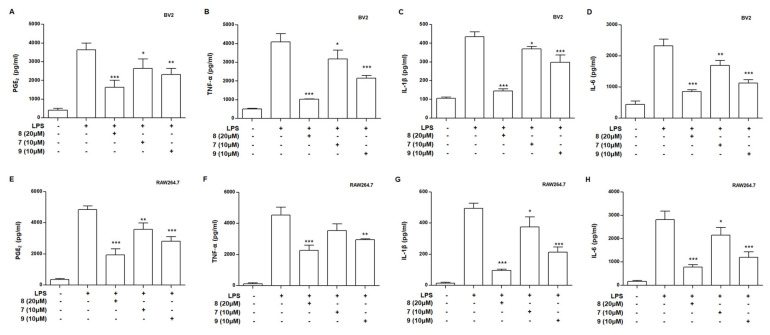
Effects of compounds 7–9 on levels of PGE_2_ (**A**,**E**), tumor necrosis TNF-α (**B**,**F**), IL-1β (**C**,**G**), and IL-6 (**D**,**H**) in lipopolysaccharide (LPS)-stimulated BV2 and RAW264.7 cells. Cells were pretreated for 3 h with indicated concentrations of compounds **7**–**9** and stimulated for 24 h with LPS (1 μg/mL). PGE_2_ assay and ELISA analysis were performed as described in Materials and Methods. Bars represent means ± standard deviation of three independent experiments. * *p* < 0.05, ** *p* < 0.01, *** *p* < 0.001 compared with LPS-treated group.

Macrophages and microglia play an important role in maintaining homeostasis of our body, they can be activated by many stimulates, such as ROS, external stimuli, and pro-inflammatory mediators. Cytokines have a complex regulatory action on inflammatory and immune responses. Pro-inflammatory cytokines, which contain both IL-6 and TNF-α, are mainly produced by various types of activated immune cells and are associated with all inflammatory properties of inflammatory diseases [26]. Therefore, we assessed the inhibitory effects of compounds **7**–**9** on the LPS-induced production of NO, PGE_2_, TNF-α, IL-1β, and IL-6. BV2 and RAW264.7 cells were incubated with compounds **7**–**9** for 3 h and then stimulated with LPS for 24 h. The production of PGE_2_, TNF-α, IL-1β, and IL-6 was significantly inhibited by compounds **7**–**9** in BV2 cells (Figure 5A–D). Figure 5E–H show that compounds **7**–**9** also significantly repressed PGE_2_, TNF-α, IL-1β, and IL-6 in BV2 cells. These results suggest that compounds **7**–**9** exert anti-neuroinflammatory and anti-inflammatory effects by modulating inflammatory mediators and cytokines.

### 3.5. Effects of Compounds **7**–**9** on the Protein Expression Levels of Inducible Nitric Oxide Synthase (iNOS) and Cyclooxygenase-2 (COX-2) in LPS-Stimulated BV2 and RAW264.7 Cells

**Figure 6:**
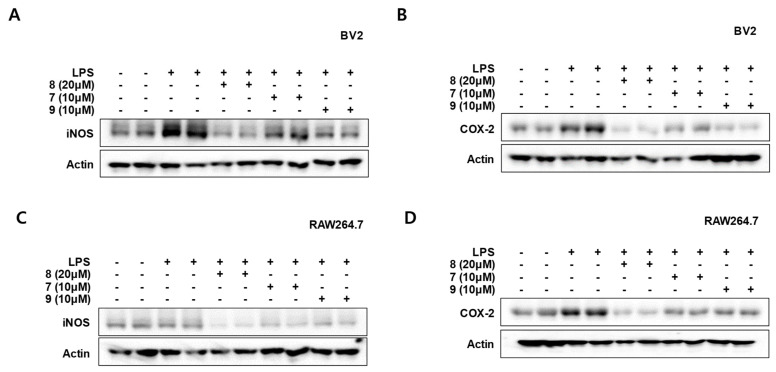
Protein expression levels of inducible nitric oxide synthase (iNOS) and cyclooxygenase-2 (COX-2) by compounds **7**–**9** in LPS-stimulated BV2 (**A**,**B**) and RAW264.7 (**C**,**D**) cells. Cells were pretreated for 3 h with indicated concentrations of compounds **7**–**9** and stimulated for 24 h with LPS (1 μg/mL). Western blot analysis was performed as described in Materials and Methods. Representative blots from three independent experiments are shown.

Inflammatory cytokines and mediators are rapidly increased after macrophages and microglia are activated. The expression of pro-inflammatory proteins can also be increased. In BV2 and RAW264.7 cells, LPS induces NO production through activating iNOS expression. LPS also increases COX-2 expression, which mediates the synthesis of prostaglandins and cytokines. NO or prostaglandins regulate various biological functions of immune responses [27]. Therefore, we assessed iNOS and COX-2 expression in BV2 and RAW264.7 cells that affect the production of inflammatory mediators and cytokines, respectively. Cells were pretreated for 3 h with indicated concentrations of compounds **7**–**9** and stimulated for 24 h with LPS. The LPS-induced expression of iNOS and COX-2 in both types of cells was significantly inhibited by compounds **7**–**9** (Figure 6). In this result, compounds **7**–**9** significantly regulated iNOS and COX-2 protein expression, suggesting that it exhibits inhibitory effects on inflammatory mediators and cytokines.

### 3.6. Effects of Compounds **7**–**9** on p38, c-Jun N-Terminal Kinase (JNK)-1/2, and Extracellular Signal-Regulated Kinase (ERK)-1/2 Phosphorylation in BV2 and RAW264.7 Cells

**Figure 7:**
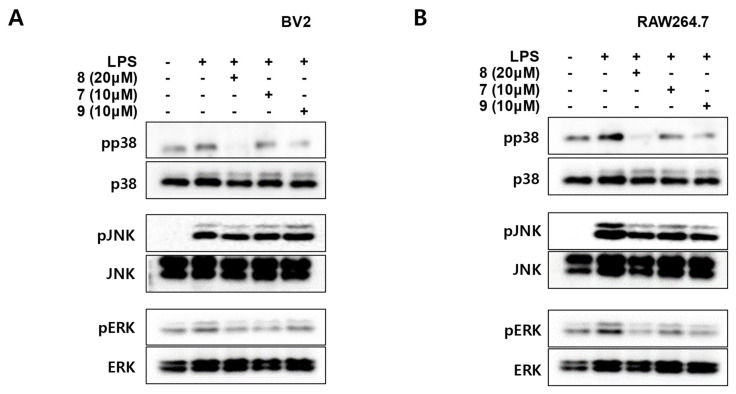
Effects of compounds **7***–***9** on p38, JNK-1/2, and ERK-1/2 phosphorylation in BV2 (**A**) and RAW264.7 (**B**) cells. Cells were pretreated with indicated concentrations of compounds **7***–***9** for 3 h and stimulated for 30 min with LPS (1 μg/mL). Cell extracts were analyzed using Western blotting with antibodies specific for phosphorylated p-p38, p-JNK1/2, and p-ERK1/2. Membranes were stripped and re-probed to measure total abundance of each mitogen-activated protein kinase (MAPK) as a control measurement. Representative blots from three independent experiments are shown.

In mammalian cells and tissues, many physiological and pathological responses are mediated by the mitogen-activated protein kinase (MAPK) signaling pathway, including stress responses, inflammation, and apoptosis. ROS modify the gene expression of pro-inflammatory mediators by altering MAPK cascades [28]. MAPKs are activated during the process, releasing ILs, TNFs, and various inflammatory mediators such as NO, PGE_2_, histamine, and lysosome granules [29]. Therefore, we explored the effects of compounds **7**–**9** on the MAPK pathway activation in BV2 and RAW264.7 cells. Cells were incubated with compounds **7**–**9** for 3 h and stimulated with LPS for 30 min. First, compound **7** inhibited the LPS-induced phosphorylation of p38 and ERK MAPKs, but did not reduce the phosphorylation of JNK in both BV2 and RAW264.7 cells (Figure 7). Compound **8** showed an excellent inhibitory effect on the LPS-induced phosphorylation of p38 and ERK MAPK in both BV2 and RAW264.7 cells, but not the phosphorylation of JNK (Figure 7). Compound **9** showed a significant inhibitory effect on LPS-induced phosphorylation of p38 MAPK and showed a tendency to inhibit ERK MAPK phosphorylation in BV2 cells. In addition, Compound **9** significantly inhibited the phosphorylation of p38 and ERK MAPK in RAW264.7 cells. However, it did not decrease the phosphorylation of JNK in both BV2 and RAW264.7 cells (Figure 7). Taken together, in both BV2 and RAW264.7 cells, compounds **7**–**9** (acanthosessilioside K, L, and M) had inhibitory effects on the phosphorylation of p38 and ERK MAPKs, but not the phosphorylation of JNK. In addition, compound **8** (acanthosessilioside L) had the most effects on the inhibition of MAPK phosphorylation. These results indicate that acanthosessilioside L significantly regulated the inflammatory mediators and cytokines through the inhibiting p38 and ERK MAPK.

## 4. Conclusions

Nine noble 3,4-seco-triterpenoid glycosides, acanthosessilioside G–O, along with two previously known triterpenoid glycosides were isolated from *A. sessiliflorus* fruits, and the chemical structures were determined without ambiguity based on the intensive analysis of 1D-NMR, 2D-NMR, UV, IR, and MS data. In this study, the LC–MS/MS MRM analysis method for quality control of the *A. sessiliflorus* fruit was first developed using the isolated compounds **1**–**11**. The advantages of hybrid LC–QTOF mass spectrometry include not only accurate and sensitivity, but also fast LC–MS/MS MRM analysis, making structural elucidations easier. It can be used for the qualitative and quantitative determination of minor or novel compounds, which is helpful in improving the quality control of *A. sessiliflorus* fruits. Acanthosessilioside K, L, and M (**7***–***9**) were the most effective in inhibiting NO, PGE_2_, TNF-α, IL-1β, and IL-6 production and reducing iNOS and COX-2 expression. In addition, these compounds had inhibitory effects on p38 and ERK MAPK phosphorylation in both BV2 and RAW264.7 cells. Our results suggest that acanthosessilioside K, L, and M could be good candidates for the development of therapeutic agents for inflammatory and neuroinflammatory diseases.

## Figures and Tables

**Table 1:** ^1^H-NMR data of acanthosessilioside G–O.

No.	δ_H_, Coupling Pattern, *J* in Hz^a)^				
	G (1)	H (2)	I (3)	J (5)	K (7)
1	1.82, overlap	1.91, overlap	1.91, overlap	2.17, overlap 1.54, overlap	3.70, overlap
2	1.39, overlap 1.36, overlap	1.37, overlap 1.33, overlap	1.37, overlap 1.33, overlap	1.80, overlap 1.24, overlap	2.86, overlap 2.58, overlap
9	1.73, overlap	1.77, overlap	1.77, overlap	1.75, overlap	2.63, d, 9.2
11	1.47, overlap	1.51, overlap	1.51, overlap	1.25, overlap	4.55, overlap
18	2.69, overlap	2.69, overlap	2.69, overlap	1.84, overlap	2.51, overlap
19	3.70, m	3.57, m	3.60, m	3.48, m	3.52, m
22	4.83, overlap	4.83, overlap	4.84, overlap	1.73, overlap 1.36, overlap	4.80, overlap
23	5.12, d, 2.0 4.90, d, 2.0	5.04, d, 2.0 4.81, d, 2.0	5.00, d, 2.0 4.81, d, 2.0	4.94, br. s 4.78, br. s	5.00, d, 2.0 4.80, d, 2.0
24	1.71, s	1.81, s	1.71, s	1.71, s	1.74, s
25	1.14, s	1.22, s	1.15, s	1.03, s	1.17, s
26	1.32, s	1.24, s	1.17, s	1.13, s	1.19, s
27	0.81, s	0.88, s	0.79, s	0.77, s	0.82, s
29	2.13, s	1.99, s	1.95, s	1.72, s	1.95, s
30a	4.90, overlap 4.79, overlap	4.95, overlap 4.89, overlap	4.90, overlap 4.81, overlap	4.79, overlap 4.75, overlap	4.96, overlap 4.89, overlap
OCH_3_	-	-	3.63, s	3.64, s	-
1′	6.37, d, 8.0	6.38, d, 8.0	6.32, d, 8.0	6.31, d, 8.0	6.33, d, 8.0
2′	4.30, overlap	4.09, overlap	4.10, overlap	4.09, overlap	4.10, overlap
3′	4.37, overlap	4.38, overlap	4.32, overlap	4.38, overlap	4.35, overlap
4′	4.30, overlap	4.30, overlap	4.28, overlap	4.30, overlap	4.29, overlap
5′	4.00, overlap	4.13, overlap	4.14, overlap	4.13, overlap	4.15, overlap
6′	4.58, dd, 12.0, 2.4 4.46, dd, 12.0, 4.8	4.69, overlap 4.30, overlap	4.64, overlap 4.30, overlap	4.69, overlap 4.30, overlap	4.66, overlap 4.29, overlap
1′′	5.35, d, 8.0	4.94, d, 8.0	4.83, d, 8.0	4.96, d, 8.0	4.94, d, 8.0
2′′	4.07, dd, 8.0, 8.0	4.10, overlap	4.10, overlap	4.10, overlap	4.10, overlap
3′′	4.27, overlap	4.25, overlap	4.25, overlap	4.25, overlap	4.25, overlap
4′′	4.30, overlap	4.38, overlap	4.31, overlap	4.38, overlap	4.35, overlap
5′′	4.00, overlap	4.12, overlap	4.15, overlap	4.12, overlap	4.15, overlap
6′′	4.42, dd, 12.0, 2.0 4.34, dd, 12.0, 5.2	4.19, overlap 4.09, overlap	4.21, overlap 4.08, overlap	4.19, overlap 4.09, overlap	4.18, overlap 4.06, overlap
1‴	-	5.83, br. s	5.75, br. s	5.79, br. s	5.79, br. s
2‴	-	4.66, overlap	4.61, overlap	4.66, overlap	4.66, overlap
3‴	-	4.53, dd, 8.4, 3.2	4.47, dd, 8.4, 3.2	4.53, dd, 8.4, 3.2	4.55, dd, 8.4, 3.2
4‴	-	3.93, dd, 8.4, 8.4	3.88, dd, 8.4, 8.4	3.93, dd, 8.4, 8.4	3.89, dd, 8.4, 8.4
5‴	-	4.93, overlap	4.92, overlap	4.93, overlap	4.93, overlap
6‴	-	1.71, d, 6.0	1.65, d, 6.0	1.67, d, 6.4	1.67, d, 6.0
1⁗					4.42, overlap
2⁗					2.05, overlap
3⁗					0.79, overlap
**No.**	**δ_H_, Coupling Pattern, *J* in Hz^a)^**				
	**L (8)**	**M (9)**	**N (10)**	**O (11)**	
1	4.86, dd, 12.0, 2.4	4.86, dd, 12.0, 2.8	4.87, overlap	4.86, overlap	
2	3.65, overlap 2.66, dd, 12.0, 8.0	3.65, dd, 12.0, 2.8 2.66, dd, 12.0, 8.0	3.65, overlap 2.66, overlap	3.65, overlap 2.64, overlap	
9	1.88, d, 11.2	1.90, d, 10.8	1.95, overlap	1.95, overlap	
11	1.25, overlap	4.07, overlap	4.07, overlap	4.07, overlap	
18	1.72, overlap	1.80, dd, 12.0, 11.2	1.72, overlap	1.72, overlap	
19	3.50, m	3.34, m	3.58, m	3.49, m	
22	2.45, overlap 1.42, overlap	2.35, overlap 1.44, overlap	4.80, overlap	4.79, overlap	
23	1.24, s	1.16, s	1.24, s	4.93, d, 2.0 4.80, d, 2.0	
24	1.37, s	1.39, s	1.37, s	1.37, s	
25	1.32, s	1.31, s	1.33, s	1.32, s	
26	1.21, s	1.15, s	1.21, s	1.24, s	
27	1.10, s	1.13, s	1.15, s	1.15, s	
29	1.93, s	1.68, s	1.96, s	1.93, s	
30	4.91, br. s 4.66, br. s	4.80, br. s 4.64, overlap	4.83, overlap 4.63, overlap	4.87, overlap 4.66, overlap	
OCH_3_	3.60, s	3.58, s	3.60, s	3.60, s	
1′	6.37, d, 8.4	6.29, d, 8.4	6.31, d, 8.4	6.31, d, 8.0	
2′	4.15, overlap	4.16, overlap	4.16, overlap	4.17, overlap	
3′	4.27, overlap	4.27, overlap	4.27, overlap	4.28, overlap	
4′	4.32, overlap	4.34, overlap	4.33, overlap	4.32, overlap	
5′	4.10, overlap	4.09, overlap	4.08, overlap	4.09, overlap	
6′	4.41, br. d, 12.0 4.38, dd, 12.0, 6.0	4.66, overlap 4.29, overlap	4.67, overlap 4.29, overlap	4.66, overlap 4.30, overlap	
1′′	4.92, d, 8.0	4.92, d, 7.6	4.90, d, 8.0	4.90, d, 8.0	
2′′	4.12, overlap	4.10, overlap	4.10, overlap	4.10, overlap	
3′′	4.25, overlap	4.25, overlap	4.25, overlap	4.25, overlap	
4′′	4.35, overlap	4.34, overlap	4.33, overlap	4.34, overlap	
5′′	4.15, overlap	4.16, overlap	4.16, overlap	4.17, overlap	
6′′	4.19, overlap 4.07, overlap	4.19, overlap 4.05, overlap	4.18, overlap 4.06, overlap	4.18, overlap 4.03, overlap	
1‴	5.85, br. s	5.79, d, 1.2	5.78, br. s	5.77, br. s	
2‴	4.68, overlap	4.64, overlap	4.63, overlap	4.62, overlap	
3‴	4.54, dd, 8.4, 3.2	4.49, dd, 8.4, 3.2	4.48, dd, 8.4, 3.2	4.49, dd, 8.4, 3.2	
4‴	3.91, dd, 8.4, 8.4	3.89, dd, 8.4, 8.4	3.88, dd, 8.4, 8.4	3.88, dd, 8.4, 8.4	
5‴	4.93, overlap	4.88, overlap	4.92, overlap	4.92, overlap	
6‴	1.69, d, 6.0	1.67, d, 6.0	1.66, d, 6.0	1.64, d, 6.0	
1⁗	-	4.37, overlap	-	-	
2⁗	-	2.01, overlap	-	-	
3⁗	-	0.76, overlap	-	-	

^1^H-NMR spectrum was measured in pyridine-*d*_5_ at 400 MHz^a)^.

**Table 2:** ^13^C-NMR data of acanthosessilioside G–O.

No.	δ_C_^a)^								
	G (1)	H (2)	I (3)	J (5)	K (7)	L (8)	M (9)	N (10)	O (11)
1	35.1	35.8	34.7	30.9	34.8	87.1	87.3	87.2	69.7
2	29.7	29.9	28.6	30.1	29.9	41.8	38.7	41.9	37.6
3	173.6	175.2	174.3	178.5	174.8	173.4	173.0	173.4	175.2
4	148.1	148.5	147.9	148.6	148.0	79.3	79.2	79.2	148.4
5	50.6	50.8	50.3	50.3	50.4	55.9	56.1	56.1	51.4
6	27.4	27.1	26.6	25.9	28.9	17.9	19.2	18.8	31.0
7	33.5	33.4	32.9	34.7	33.0	35.3	35.4	35.4	33.8
8	40.1	40.0	39.5	39.5	39.6	42.8	42.9	42.9	42.4
9	41.6	41.6	41.1	41.0	41.2	48.8	48.9	49.0	49.5
10	43.7	43.7	43.2	43.2	43.2	43.9	47.0	47.0	45.9
11	22.3	22.3	21.7	21.6	64.2	22.8	67.6	67.7	64.2
12	25.4	25.5	24.9	24.9	25.9	37.2	37.0	37.3	36.7
13	38.9	38.7	38.2	38.4	38.3	38.5	37.5	38.5	37.4
14	41.2	41.3	40.8	40.8	40.8	42.7	42.8	42.8	43.0
15	30.2	30.1	29.6	28.6	31.0	29.8	30.4	29.8	32.6
16	26.3	26.4	25.9	32.2	26.6	32.5	31.1	32.6	32.2
17	63.3	63.6	63.1	57.0	63.1	49.6	57.0	58.1	57.1
18	44.6	44.7	44.2	49.8	44.3	47.4	49.5	49.6	49.7
19	48.3	48.0	47.5	47.3	47.6	46.9	47.3	47.4	47.8
20	152.1	151.7	151.2	150.8	151.2	150.8	150.5	150.9	150.4
21	42.6	42.4	41.9	32.9	41.9	30.6	30.9	30.2	41.7
22	76.0	75.7	76.4	36.8	76.5	36.8	36.7	76.4	76.5
23	114.4	114.0	113.6	113.7	113.7	24.8	24.9	24.9	113.9
24	23.9	24.1	23.4	23.4	23.4	32.4	32.6	32.5	23.7
25	20.7	21.0	20.3	20.3	20.4	18.5	18.8	19.2	20.9
26	16.8	16.8	16.3	16.3	16.3	17.9	17.9	17.9	17.4
27	15.3	15.3	14.8	14.7	14.9	15.1	15.2	15.2	14.8
28	179.0	177.0	174.8	174.9	174.0	174.7	174.9	174.7	175.0
29	19.7	19.7	19.2	19.5	19.3	19.2	19.6	19.3	19.6
30	110.9	111.0	110.5	110.0	110.6	110.9	110.2	110.7	110.0
OCH_3_	-	-	51.3	51.3	-	51.1	49.6	51.0	51.1
1′	94.3	95.8	95.3	95.3	95.4	95.4	95.3	95.4	95.4
2′	83.6	74.5	73.9	74.0	74.0	73.9	74.0	73.9	74.0
3′	78.6	77.5	77.9	78.0	78.0	77.9	78.1	78.2	78.1
4′	71.1	71.4	70.3	70.3	71.0	70.8	71.0	71.0	71.1
5′	79.2	78.8	78.4	78.4	78.4	78.0	78.5	78.4	78.5
6′	62.5	70.0	69.5	69.5	69.6	69.5	69.5	69.6	69.6
1′′	107.1	105.6	105.0	105.1	105.1	105.1	105.1	105.1	105.1
2′′	76.8	75.4	74.0	74.1	74.1	74.0	74.1	74.1	74.1
3′′	78.5	78.4	77.0	77.0	77.1	77.1	77.1	77.1	77.2
4′′	71.8	76.9	76.4	76.5	76.5	76.3	76.5	76.4	76.5
5′′	79.7	79.1	78.6	78.7	78.7	78.6	78.7	78.7	78.7
6′′	62.4	61.8	61.3	61.4	61.4	61.1	61.4	61.4	61.4
1‴	-	103.2	102.6	102.7	102.7	102.6	102.7	102.7	102.8
2‴	-	72.9	72.5	72.5	72.5	72.5	72.5	72.5	72.5
3‴	-	73.1	72.7	72.7	72.7	72.7	72.7	72.7	72.8
4‴	-	74.4	75.2	75.3	75.2	75.2	75.3	75.2	75.3
5‴	-	70.7	70.9	71.0	70.3	70.2	70.3	70.3	70.3
6‴	-	18.9	18.4	18.5	18.4	18.5	18.4	18.4	18.5
1⁗	-	-	-	-	69.5	-	69.4	-	-
2⁗	-	-	-	-	19.4	-	19.3	-	-
3⁗	-	-	-	-	13.7	-	13.7	-	-

^13^C-NMR spectrum was measured in pyridine-*d*_5_ at 100 MHz^a)^.

**Table 3:** LC–MS/MS MRM conditions for the simultaneous quantification of acanthosessilioside **G**–**O**.

Acanthosessiliosides (No.)	MRM ^a^	Time ^b^	DP ^c^	EP ^d^	CEP ^e^	CE ^f^	CXP ^g^
G (1)	809.364/467.4	150	–55	–10.5	–36	–46	–6
H (2)	955.436/485.4	150	–60	–11	–42	–50	–6
I (3)	969.464/499.4	150	–70	–10	–26	–48	–6
J (5)	953.473/483.3	150	–75	–10	–42	–56	–8
K (7)	1011.509/541.5	150	–110	–10.5	–28	–44	–6
L (8)	969.431/499.4	150	–75	–9.5	–38	–52	–6
M (9)	1027.45/557.4	150	–75	–10.5	–42	–54	–8
N (10)	1001.44/531.4	150	–70	–8.5	–26	–46	–6
O (11)	1001.45/531.5	150	–70	–12	–46	–50	–6

^a^ MRM: multiple reaction monitoring. ^b^ Mass scan time. ^c^ DP: declustering potential. ^d^ EP: entrance potential. ^e^ CEP: collision cell entrance potential. ^f^ CE: collision energy. ^g^ CXP: collision cell exit potential.

**Table 4:** UPLC–MS/MS data-negative ion mode for acanthosessilioside G–O identified in ASFEx.

Acanthosessilioside	Molecular Formula	MW	Measured Value ^a^ [M–H]	MS/MS Fragmentation ^a^
G	C_42_H_66_O_15_	810.44	809.2	647.2 [M–H–Glc]^–^; 587.2 [M–Glu–C_2_H_3_O_2_]^–^; 485.2 [M–H–Glc–Glc]^–^; 467.4 [M–H–Glc–Glc–H_2_O]^–^; 423.0 [M–H–Glc–Glc–HCOO–H_2_O]^–^; 405.4 [M–H–Glc–Glc–HCOO– 2H_2_O]^–^; 179.2 [M–H–Glu–3,4-seco-Triterpenoid–C_2_H_3_O_2_–2C_3_H_5_–H_2_O–HCOO]^–^; 161.2[M–H–3,4-seco-Triterpenoid–Glu–C_2_H_3_O_2_–2C_3_H_5_–2H_2_O-HCOO]^–^
H	C_48_H_76_O_19_	956.50	955.6	485.4 [M–H–Rha–Glc–Glc]^–^; 469.4 [M–H–Rha–Glc–Glc–H_2_O]^–^; 439.4 [M–H–Rha–Glc–Glc–HCOO]^–^; 423.4 [M–H–Rha–Glc–Glc–HCOO–H_2_O]^–^; 405.2 [M–H–Rha–Glc–Glc–HCOO–2H_2_O]^–^; 367.0 [M–H–Rha–Glc–Glc–HCOO–H_2_O–2C_3_H_5_]^–^; 325.0 [M–H–Rha-3,4-seco-Triterpenoid–C_2_H_3_O_2_–H_2_O–2C_3_H_5_–HCOO]^–^; 161.2 [M–H–Rha–Glu–3,4-seco-Triterpenoid – C_2_H_3_O_2_–H_2_O–2C_3_H_5_–HCOO]^–^
I	C_49_H_78_O_19_	970.51	969.6	808.0 [M–H–Rha]^–^; 499.4 [M–H–Rha–Glc–Glc]^–^; 469.2 [M–H–3,4-seco-Triterpenoid –C_3_H_5_O_2_–2C_3_H_5_–H_2_O–HCOO]^–^; 455.0 [M–H–Rha–Glc–Glc–HCOO]^–^; 365.4 [M–H–Rha–Glc–Glc–HCOO–C_3_H_5_O_2_–H_2_O]^–^; 325.0 [M–H–Rha–3,4-seco-Triterpenoid–C_3_H_5_O_2_–2C_3_H_5_–H_2_O–HCOO]^–^; 160.6 [M–H–Rha–Glu–3,4-seco-Triterpenoid–C_3_H_5_O_2_–2C_3_H_5_–2H_2_O–HCOO]^–^
J	C_49_H_78_O_18_	954.52	953.6	483.2 [M–H–Rha–Glc–Glc]^–^; 469.2 [M–H–Rha–Glc–Glc–H_2_O]^–^; 409.2 [M–H–Rha–Glc–Glc–HCOO–C_3_H_6_O_2_]^–^; 367.2 [M–H–Rha–Glc–Glc–HCOO–C_3_H_6_O_2_–C_3_H_5_]^–^; 323.4 [M–H–Rha–Glc–Glc–HCOO–C_3_H_6_O_2_–C_3_H_5_–HCOO]^-^; 160.8 [M–H–Rha–Glu–3,4-seco-Triterpenoid–C_3_H_5_O_2_–2C_3_H_5_–HCOO–H_2_O]^–^
K	C_51_H_80_O_20_	1012.52	1011.5	849.6 [M–H–Rha–H_2_O]^–^; 541.4 [M–H–Rha–Glc–Glc]^–^; 479.6 [M–H–Rha–Glu–Glu–HCOO–H_2_O]^–^; 469.2 [M–H–3,4-seco-Triterpenoid–HCOO]^–^; 454.8 [M–H–Rha–Glu–Glu–HCOO–C_3_H_5_]^–^; 405.4 [M–H–Rha–Glu–Glu–C_2_H_2_O_2_–C_3_H_7_O–H_2_O]^–^; 364.8 [M–H–Rha–Glu–Glu–C_2_H_2_O_2_–C_3_H_7_O–H_2_O–C_3_H_5_]^–^; 325.0 [M–H–Rha–Glu–Glu–C_2_H_2_O_2_–C_3_H_7_O–H_2_O–2C_3_H_5_]^–^; 160.8 [M–H–Rha–Glu–3,4-seco-Triterpenoid–C_2_H_2_O_2_–2H_2_O–2C_3_H_5_–C_3_H_7_O–HCOO]^–^
L	C_49_H_78_O_19_	970.51	969.6	499.4 [M–H–Rha–Glc–Glc]^–^; 469.2 [M–H–3,4-seco-Triterpenoid–HCOO]^–^; 323.0[M–H–Rha–3,4-seco-Triterpenoid–C_3_H_5_O_2_–C_3_H_5_–HCOO]^–^;160.8[M–H–Rha–Glu–3,4-seco-Triterpenoid–C_3_H_5_O_2_–C_3_H_5_–HCOO]^–^
M	C_52_H_84_O_20_	1028.56	1027.6	557.4 [M–H–Rha–Glc–Glc]^–^; 468.8 [M–H–3,4-seco-Triterpenoid]^–^; 367.2 [M–H–Rha–Glc–Glc–C_3_H_6_O_2_–C_3_H_8_O–C_3_H_6_]^–^; 323.2 [M–H–Rha–3,4-seco-Triterpenoid–C_3_H_5_O_2_–C_3_H_7_O–C_3_H_5_–H_2_O–HCOO]^–^; 161.2 [M–H–Rha–Glu–3,4-seco-Triterpenoid–C_3_H_5_O_2_–C_3_H_7_O–C_3_H_5_–H_2_O–HCOO]^-^
N	C_49_H_78_O_21_	1002.50	1001.6	531.2 [M–H–Rha–Glc–Glc]^–^; 486.8 [M–H–Rha–Glc–Glc–HCOO]^–^; 469.2[M–H–Rha–Glu–Glu–HCOO–2H_2_O]^–^; 437.2 [M–H–Rha–Glu–Glu–2H_2_O–C_2_H_3_O_2_]^–^; 393.4 [M–H–Rha–Glu–Glu–HCOO–H_2_O–C_2_H_3_O_2_]^–^; 379.4 [M–H–Rha–Glu–Glu–HCOO–2H_2_O–C_3_H_5_O_2_]^–^; 367.4 [M–H–Rha–3,4-seco-Triterpenoid–2H_2_O–C_3_H_5_O_2_]^–^; 325.2 [M–H–3,4-seco-Triterpenoid–2H_2_O–C_3_H_5_O_2_–HCOO–Rha]^–^; 161.0[M–H–Rha–Glu–3,4-seco-Triterpenoid–2H_2_O–C_3_H_5_O_2_–HCOO]^–^
O	C_49_H_78_O_21_	1002.50	1001.6	531.4 [M–H–Rha–Glc–Glc]^–^; 469.0 [M–H–3,4-seco-Triterpenoid–C_3_H_5_O_2_–2H_2_O–2C_3_H_5_]^–^; 437.4 [M–H–Rha–Glc–Glc–C_2_H_3_O_2_–2H_2_O]^–^; 379.2 [M–H–Rha–Glu–Glu–C_3_H_5_O_2_–2H_2_O–HCOO]^–^; 367.2[M–H–Rha–3,4-seco-Triterpenoid–C_3_H_5_O_2_–3H_2_O–2C_3_H_5_]^–^; 325.2[M–H–Rha–3,4-seco-Triterpenoid–C_3_H_5_O_2_–3H_2_O–2C_3_H_5_–HCOO]^−^; 161.2[M–H–Rha–Glu–3,4-seco-Triterpenoid–C_3_H_5_O_2_–4H_2_O–2C_3_H_5_–HCOO]^–^

^a^ Mass accuracy < 5 ppm.

**Table 5:** Quantification, linear range, regression equation, coefficient of determination LOD and LOQ for LC–MS/MS MRM analysis of compounds **1**–**11** ^a^.

Compounds	Rt ^b^ (min)	Calibration Curve ^c^	*R* ^2 d^	Linear Range (μg/mL)	LOD ^e^ (ppm)	LOQ ^f^ (ppm)	Amount (mg/g)
1	6.20	y = 27.8x − 952	0.9988	0.1–2.5	0.0117	0.0357	0.010
2	6.20	y = 161x − 2.31 × 10^4^	0.9974	0.1–2.5	0.0028	0.0086	2.806
3	9.68	y = 146x − 5.45 × 10^3^	0.9982	0.1–2.5	0.0025	0.0077	0.059
4	7.77	y = 198x − 1.46 × 10^4^	0.9989	0.1–2.5	0.0014	0.0044	0.43
5	11.49	y = 203x − 1.99 × 10^4^	0.9987	0.1–2.5	0.0016	0.0049	0.026
6	10.12	y = 231x − 1.63e × 10^4^	0.9954	0.1–2.5	0.0026	0.008	0.009
7	12.75	y = 3.21x − 16.2	0.9998	0.05–2.5	0.0263	0.0799	0.023
8	9.19	y = 41.7x − 1.64 × 10^3^	0.9888	0.1–2.5	0.0237	0.0718	0.102
9	10.15	y = 97.5x − 1.02 × 10^4^	0.9965	0.1–2.5	0.0054	0.0165	0.017
10	4.67	y = 65.1x − 6.4 × 10^3^	0.9988	0.1–2.5	0.0048	0.0146	0.031
11	4.67	y = 91.9x − 1.38 × 10^4^	0.9958	0.1–2.5	0.0063	0.0193	0.016

^a^ Mean values of samples (*n* = 3). ^b^ Rt: retention time. ^c^ *y*: logarithmic value of peak area; *x*: logarithmic value of amount injected. ^d^ R^2^: linearity. ^e^ LOD: limit of detection. ^f^ LOQ: limit of quantitation.

## Data Availability

Data is contained within the article.

## Supplementary Materials

The following are available online at https://www.mdpi.com/article/10.3390/antiox10091334/s1, Figure S1: 1H, 13C NMR, Qtrap/MSMS, and HRESIMS of Acanthosessilioside G. Figure S2: 1H, 13C NMR, Qtrap/MSMS, and HRESIMS of Acanthosessilioside H. Figure S3: 1H, 13C NMR, Qtrap/MSMS, and HRESIMS of Acanthosessilioside I. Figure S4: 1H, 13C NMR, Qtrap/MSMS, and HRESIMS of Acanthosessilioside J. Figure S5: 1H, 13C NMR, Qtrap/MSMS, and HRESIMS of Acanthosessilioside K. Figure S6: 1H, 13C NMR, Qtrap/MSMS, and HRESIMS of Acanthosessilioside L. Figure S7: 1H, 13C NMR, Qtrap/MSMS, and HRESIMS of Acanthosessilioside M. Figure S8: 1H, 13C NMR, Qtrap/MSMS, and HRESIMS of Acanthosessilioside N. Figure S9: 1H, 13C NMR, Qtrap/MSMS, and HRESIMS of Acanthosessilioside O.

## Abbreviations

AFB	*Acanthopanax sessiliflorus* fruits *n*-butanol fraction
AFE	*Acanthopanax sessiliflorus* fruits ethyl acetate fraction
AFH	*Acanthopanax sessiliflorus* fruits aqueous fraction
ASFEx	*Acanthopanax sessiliflorus* fruits extraction
CC	column chromatography
DW	dry weight
EtOAc	ethyl acetate
FAB	subfractions from *Acanthopanax sessiliflorus* fruits *n*-butanol fraction
gHMBC	gradient heteronuclear multiple bond coherence
IFN- γ	interferon-gamma
LPS	lipopolysaccharide
MeOH	methanol
*n*-BuOH	*n*-butanol
NMR	nuclear magnetic resonance
NO	nitric oxide
ODS	octadecyl silica gel
RNS	reactive nitrogen species
ROS	reactive oxygen species
SiO_2_	silica gel
TLC	thin layer chromatography
TNF-α	tumor necrosis factor-alpha
UV	ultraviolet
^13^C-NMR	carbon-13 nuclear magnetic resonance
1D-NMR	one-dimensional (1D) NMR
2D-NMR	two-dimensional (2D) NMR
^1^H-NMR	proton-1 nuclear magnetic resonance
