# Supplementary material for: Noble 3,4-Seco-triterpenoid Glycosides from the Fruits of Acanthopanax sessiliflorus and Their Anti-Neuroinflammatory Effects
Source: Antioxidants (Basel). 2021 Aug 24;10(9):1334. doi: 10.3390/antiox10091334 (PMC8466647; doi:10.3390/antiox10091334)

Supplementary Materials

Noble 3,4-seco-Triterpenoid Glycosides from the Fruits of *Acanthopanax sessiliflorus* and their Anti-Neuroinflammatory Effects

Bo-Ram Choi [1,2†], Hyoung-Geun Kim [2†], Wonmin Ko [3], Linsha Dong [3], Dahye Yoon [1], Seon Min Oh [1,2], Young-Seob Lee [1], Dong-Sung Lee [3], Nam-In Baek [2], and Dae Young Lee [1,*]

[1] Department of Herbal Crop Research, National Institute of Horticultural and Herbal Science, RDA, Eumseong 27709, Republic of Korea; bmcbr@korea.kr (B.-R.C); dahyeyoon@korea.kr (D.Y); youngseoblee@korea.kr (Y.-S.L.)

[2] Graduate School of Biotechnology and Department of Oriental Medicinal Biotechnology, Kyung Hee University, Yongin 17104, Korea; zwang05@khu.ac.kr (H.-G.K.); nibaek@khu.ac.kr (N.-I.B.)

[3] College of Pharmacy, Chosun University, Dong-gu, Gwangju, Republic of Korea; rabis815@naver.com (W.K.); donglinsha011@163.com (L.D); dslee2771@chosun.ac.kr (D.-S.L.)

* Correspondence: dylee0809@gmail.com; Tel.: +82-43-871-5784

† Co-first author, these authors contributed equally to this work

Contents:

S1: ^{1}H, ^{13}C NMR, Qtrap/MSMS, and HRESIMS of Acanthosessilioside G

S2: ^{1}H, ^{13}C NMR, Qtrap/MSMS, and HRESIMS of Acanthosessilioside H

S3: ^{1}H, ^{13}C NMR, Qtrap/MSMS, and HRESIMS of Acanthosessilioside I

S4: ^{1}H, ^{13}C NMR, Qtrap/MSMS, and HRESIMS of Acanthosessilioside J

S5: ^{1}H, ^{13}C NMR, Qtrap/MSMS, and HRESIMS of Acanthosessilioside K

S6: ^{1}H, ^{13}C NMR, Qtrap/MSMS, and HRESIMS of Acanthosessilioside L

S7: ^{1}H, ^{13}C NMR, Qtrap/MSMS, and HRESIMS of Acanthosessilioside M

S8: ^{1}H, ^{13}C NMR, Qtrap/MSMS, and HRESIMS of Acanthosessilioside N

S9: ^{1}H, ^{13}C NMR, Qtrap/MSMS, and HRESIMS of Acanthosessilioside O

S1: ^{1}H, ^{13}C NMR, Qtrap/MSMS, and HRESIMS of Acanthosessilioside G

^{1}H NMR of Acanthosessilioside G

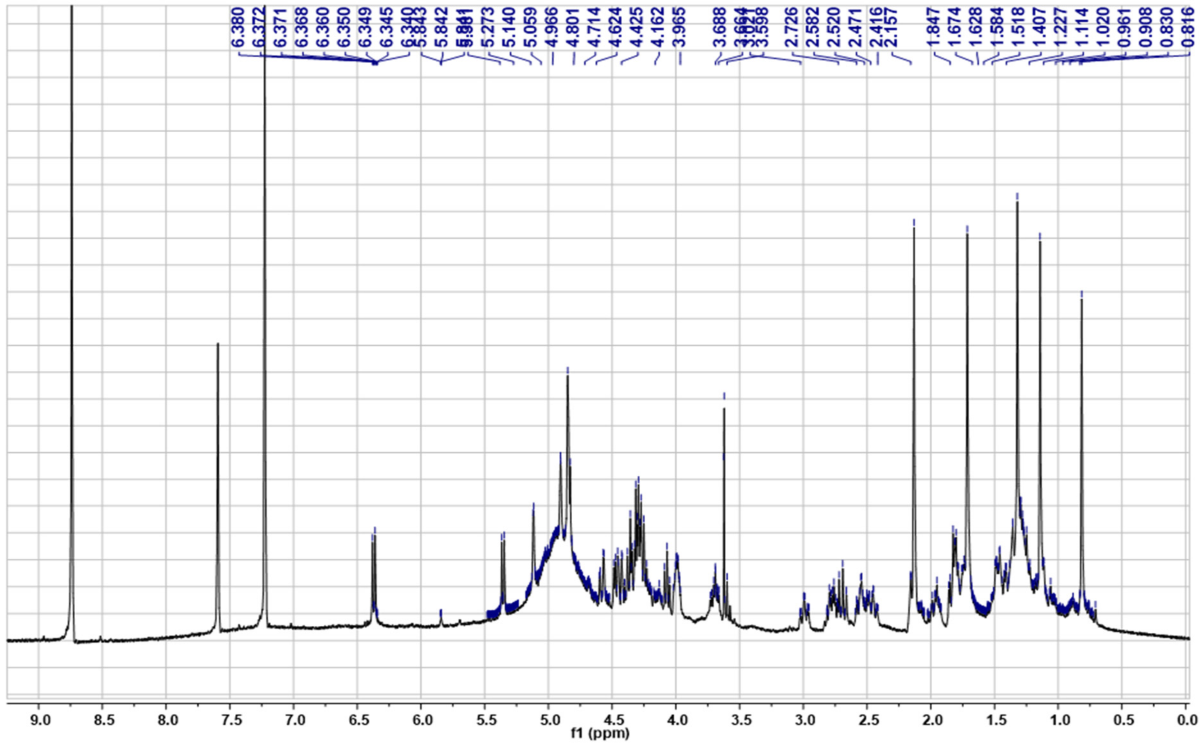

^{13}C NMR of Acanthosessilioside G

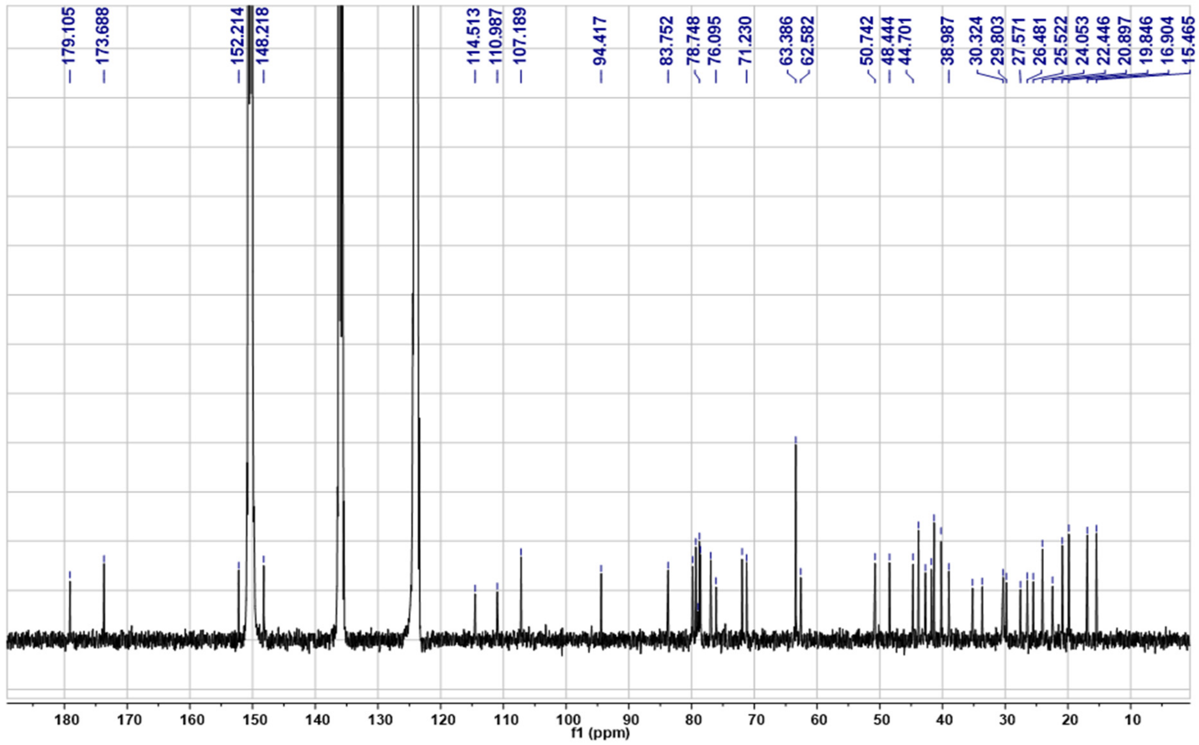

Qtrap/MSMS of Acanthosessilioside G

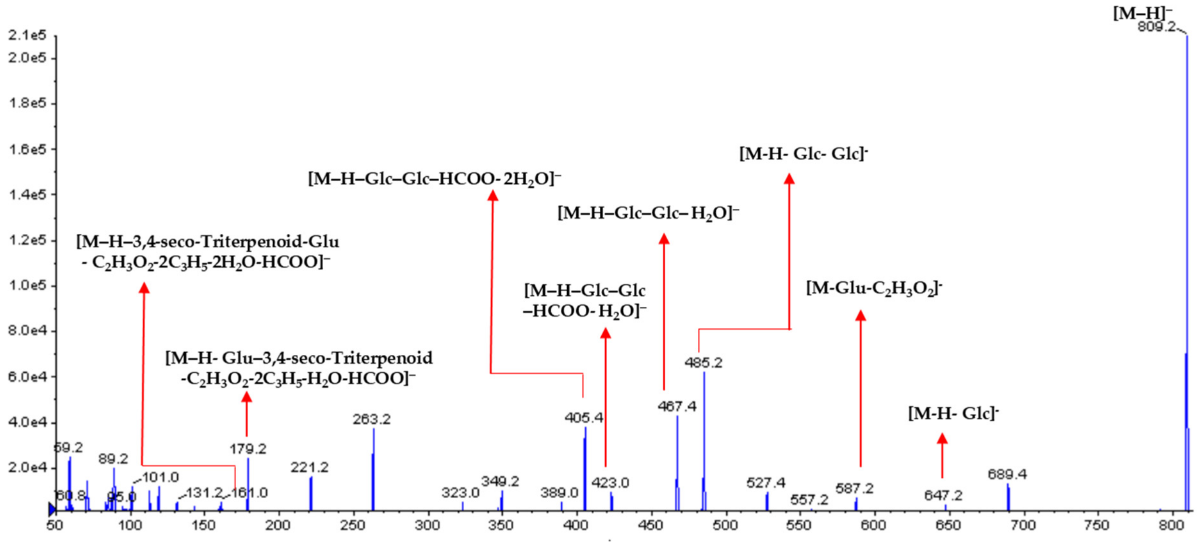

HRESIMS of Acanthosessilioside G

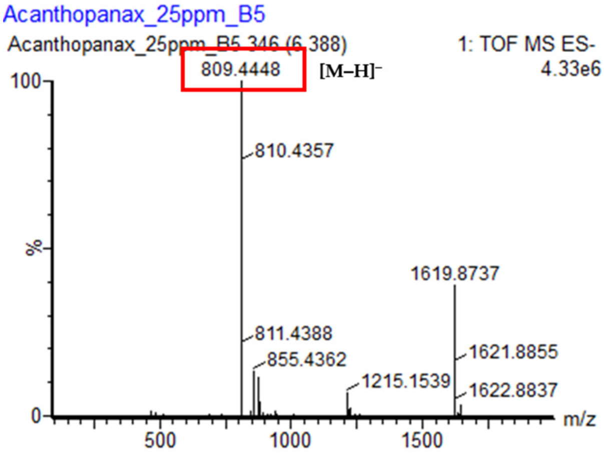

S2: ^{1}H, ^{13}C NMR, Qtrap/MSMS, and HRESIMS of Acanthosessilioside H

^{1}H NMR of Acanthosessilioside H

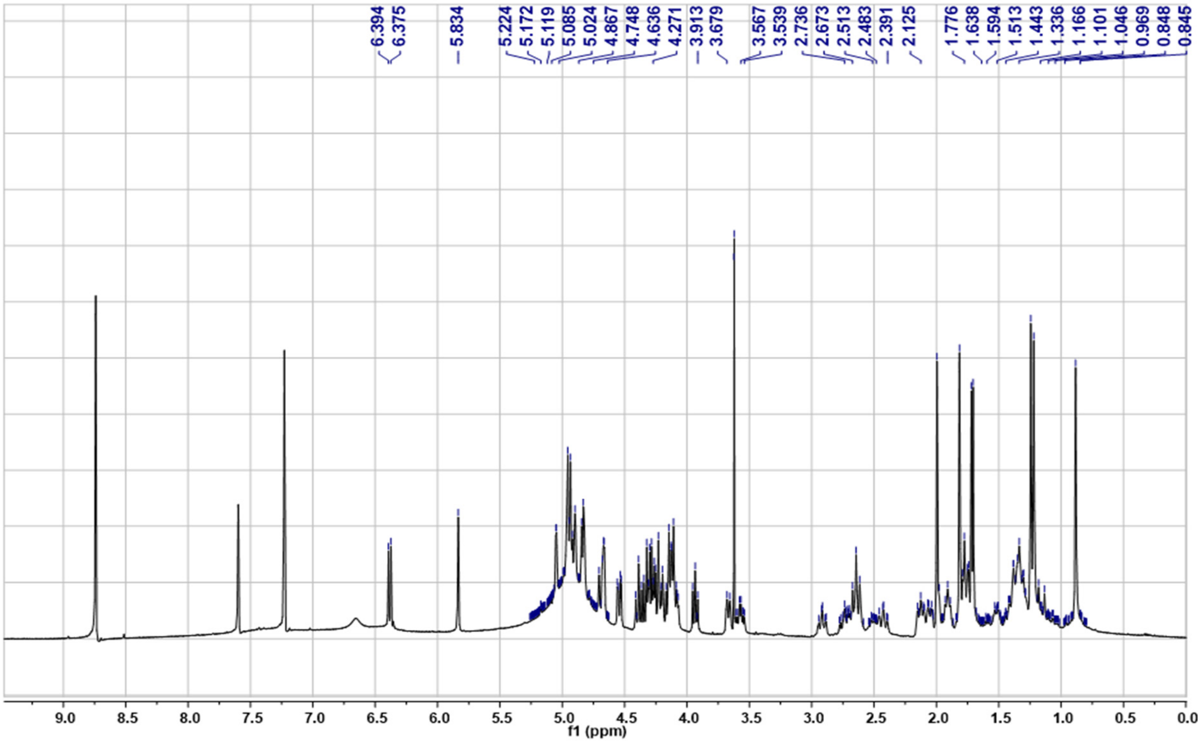

^{13}C NMR of Acanthosessilioside H

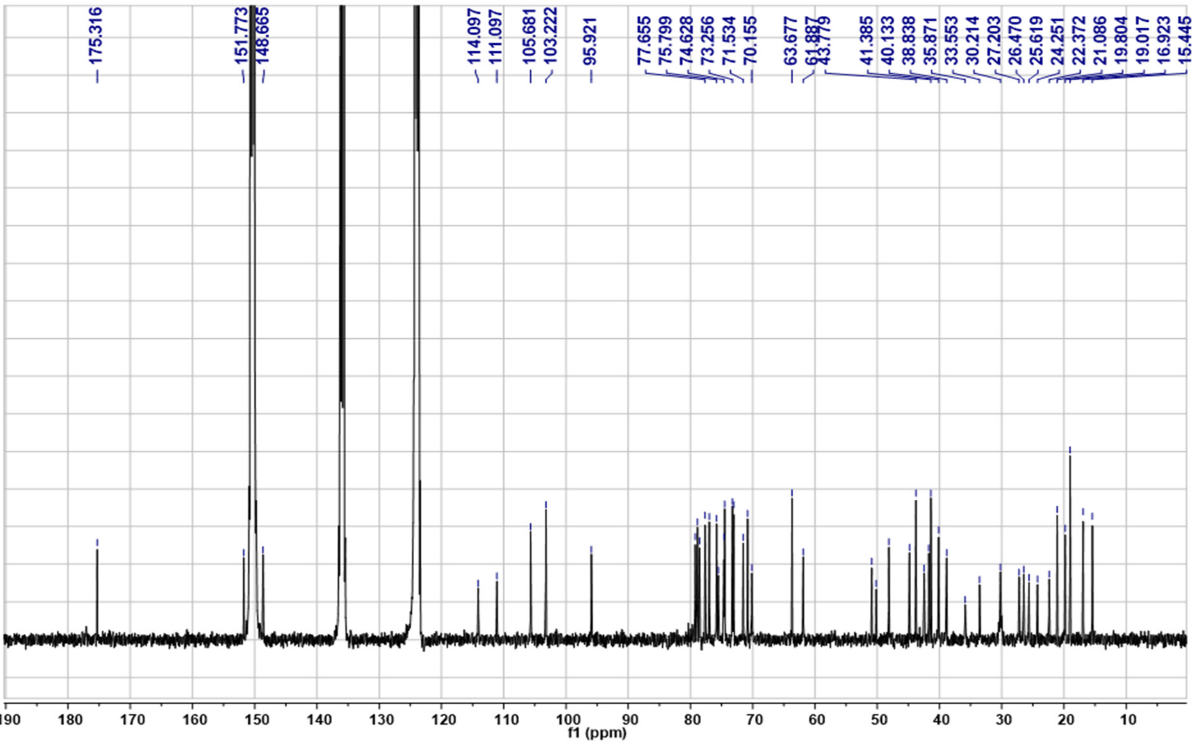

Qtrap/MSMS of Acanthosessilioside H

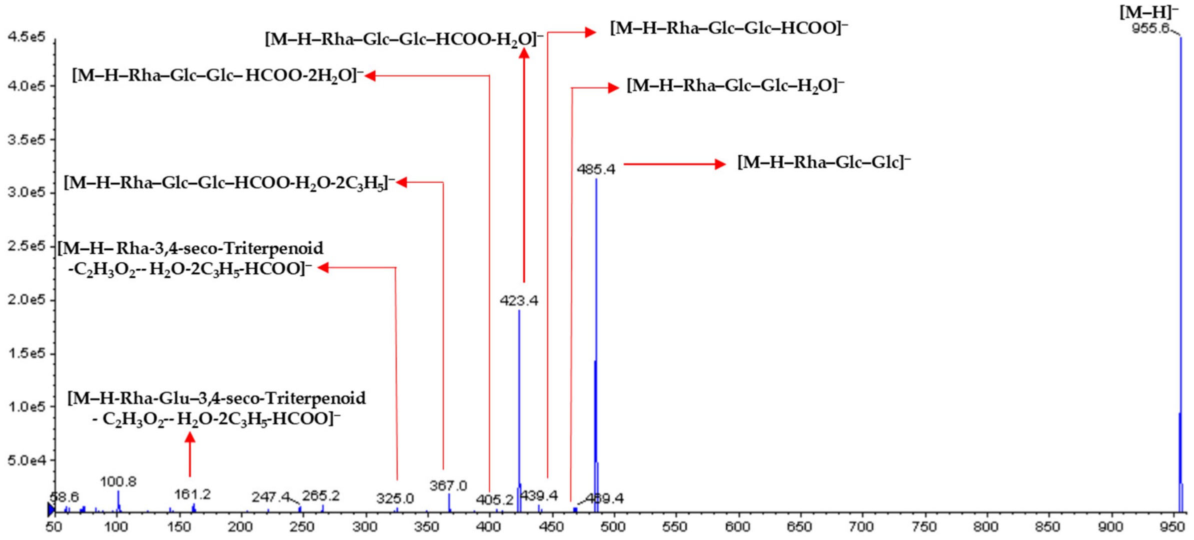

HRESIMS of Acanthosessilioside H

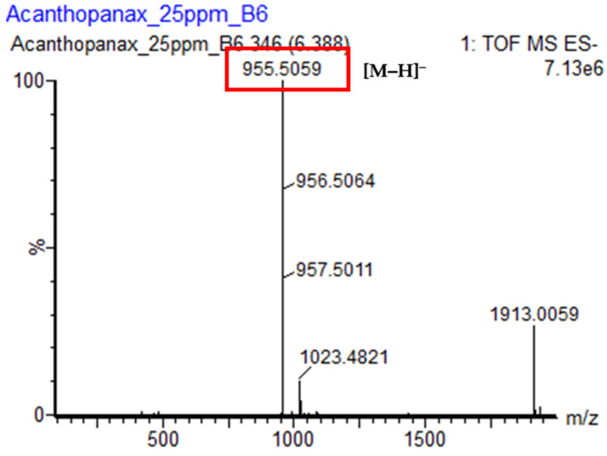

S3: ¹H, ¹³C NMR, Qtrap/MSMS, and HRESIMS of Acanthosessilioside I

¹H NMR of Acanthosessilioside I

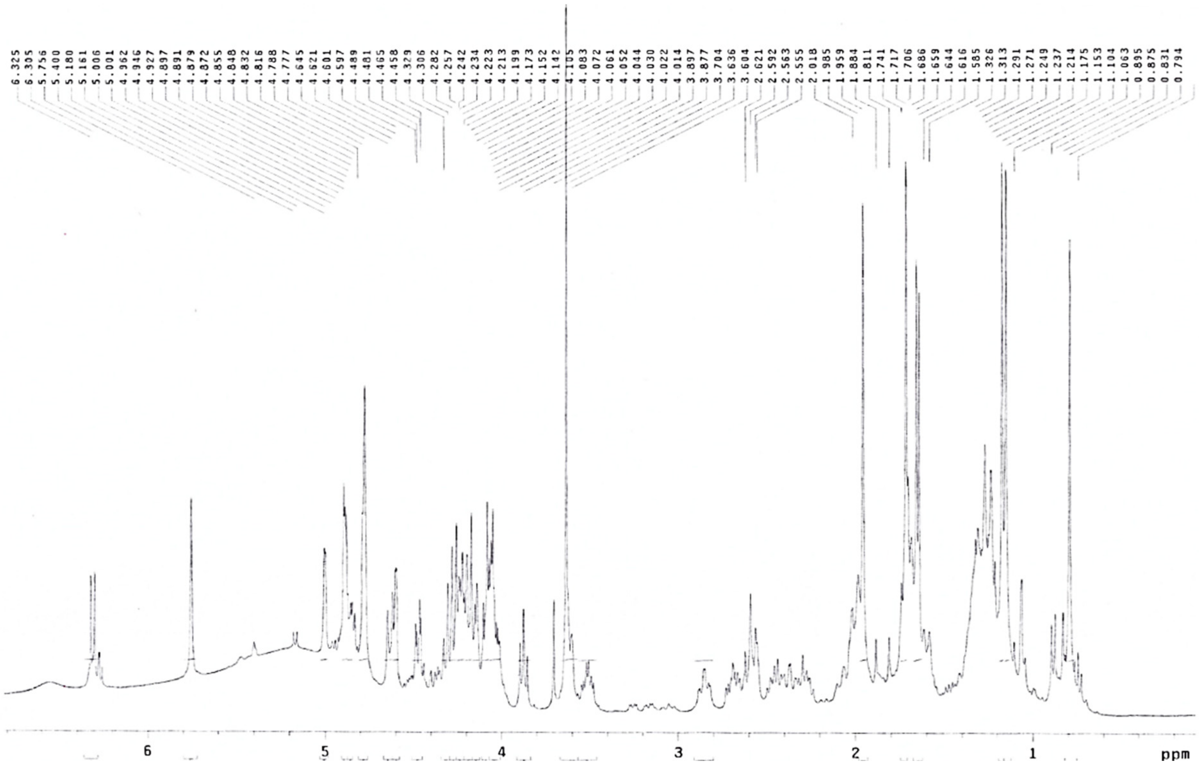

¹³C NMR of Acanthosessilioside I

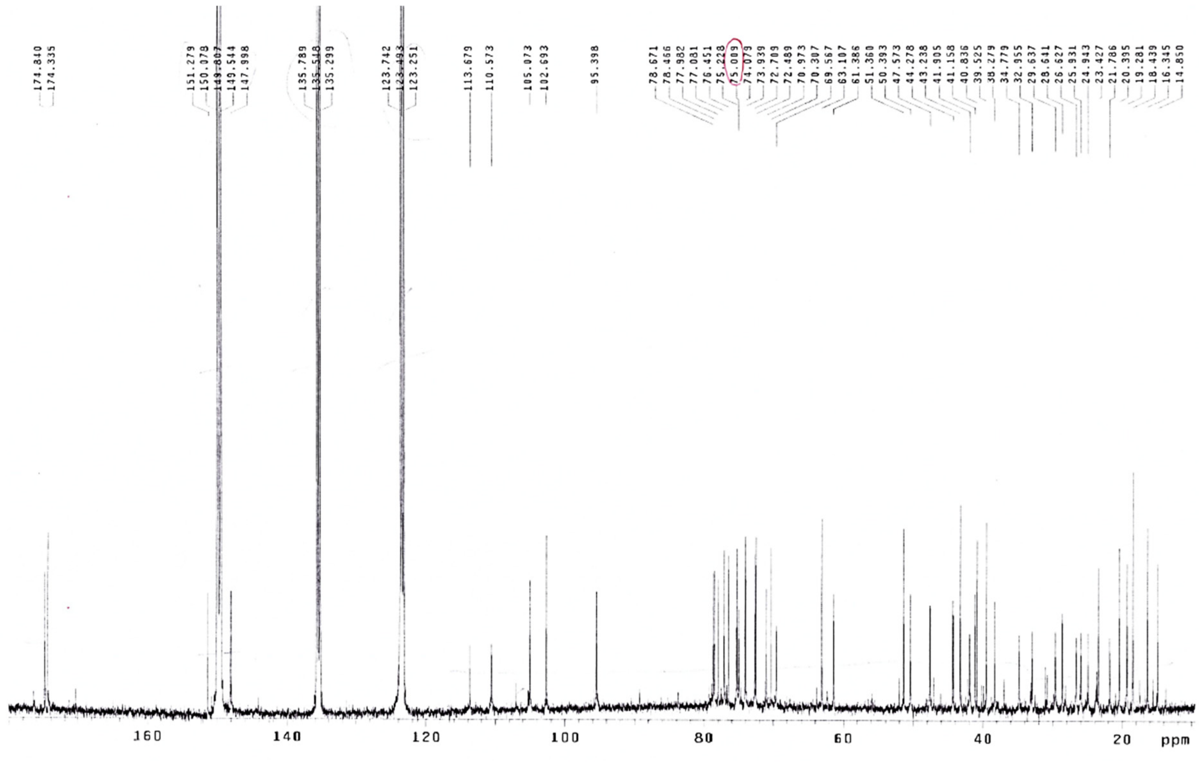

Qtrap/MSMS of Acanthosessilioside I

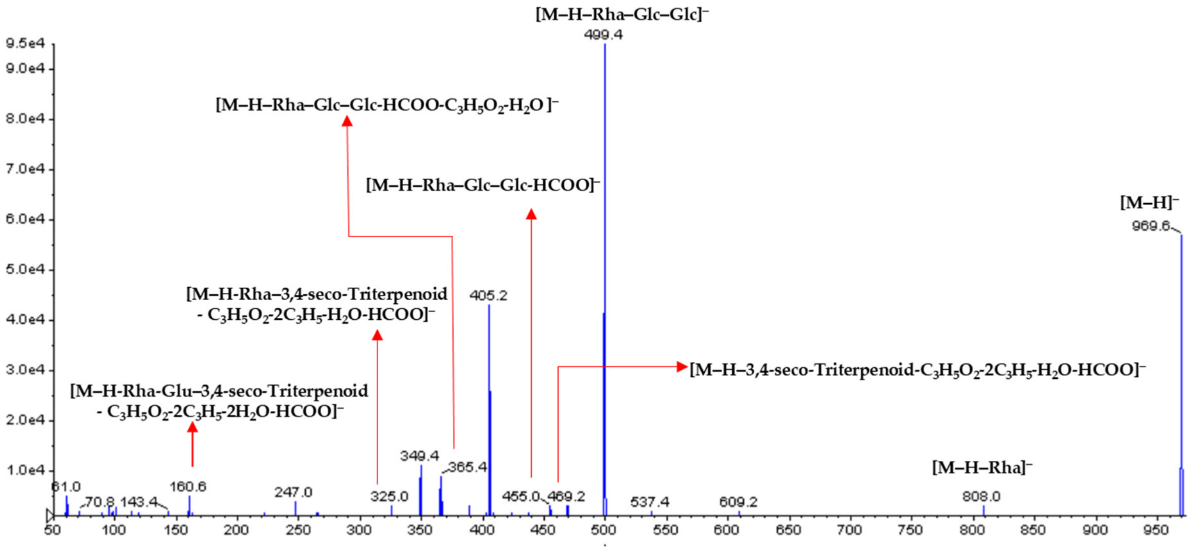

HRESIMS of Acanthosessilioside I

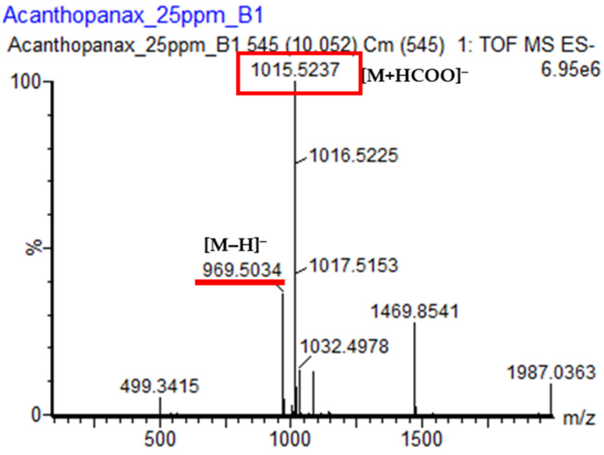

S4: ^{1}H, ^{13}C NMR, Qtrap/MSMS, and HRESIMS of Acanthosessilioside J

^{1}H NMR of Acanthosessilioside J

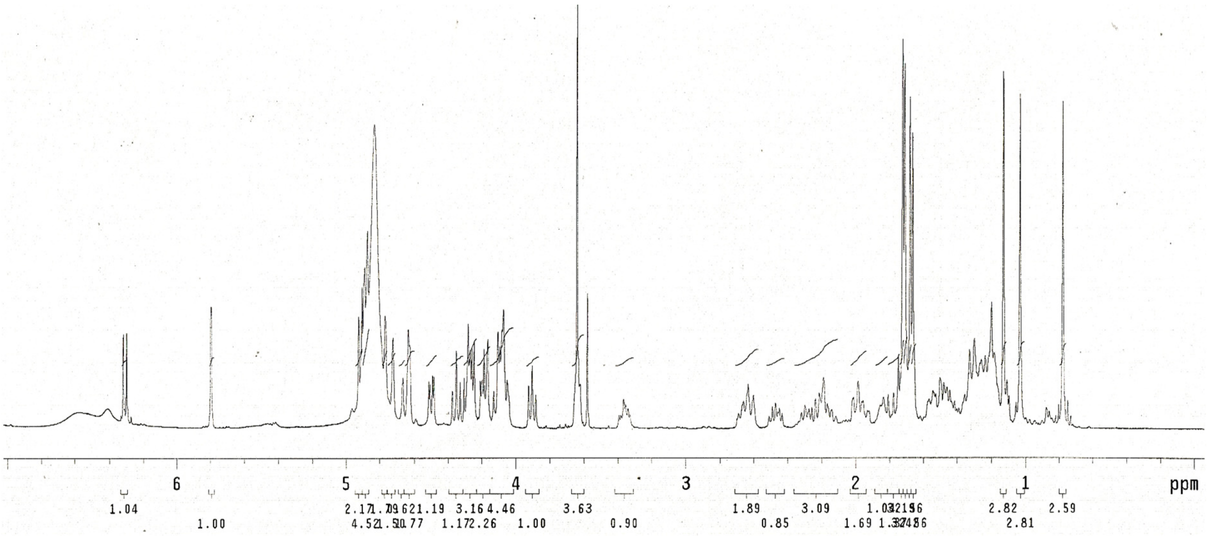

^{13}C NMR of Acanthosessilioside J

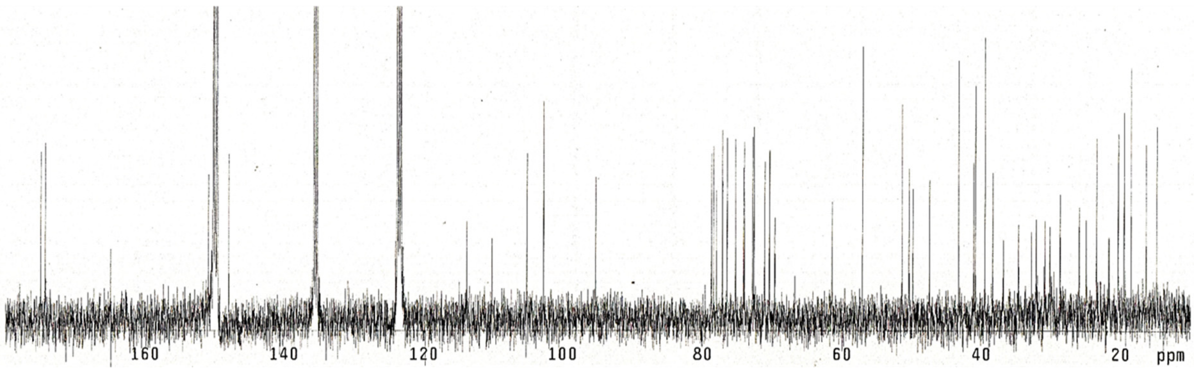

Qtrap/MSMS of Acanthosessilioside J

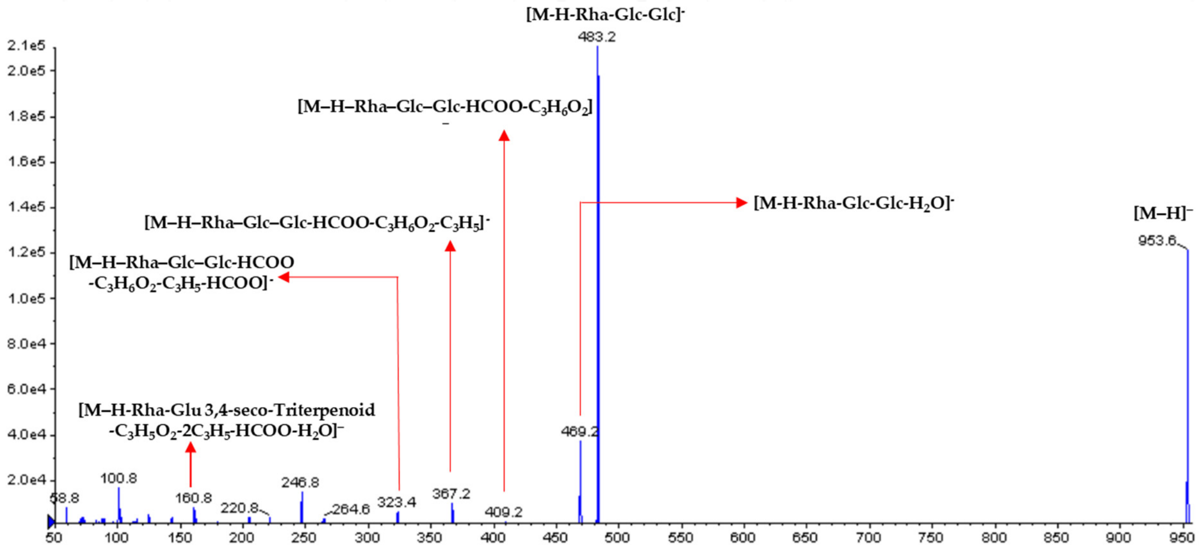

HRESIMS of Acanthosessilioside J

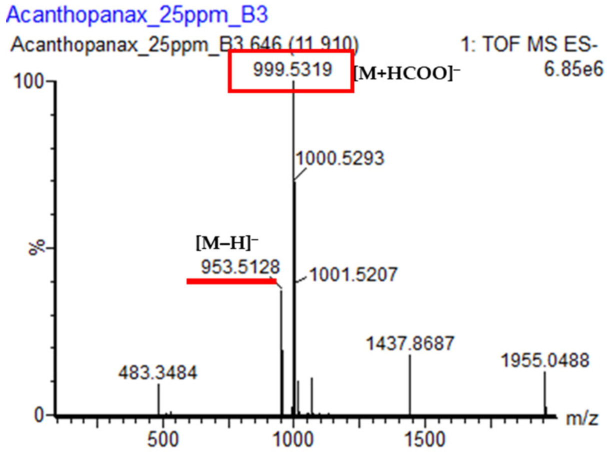

S5: ^{1}H, ^{13}C NMR, Qtrap/MSMS, and HRESIMS of Acanthosessilioside K

^{1}H NMR of Acanthosessilioside K

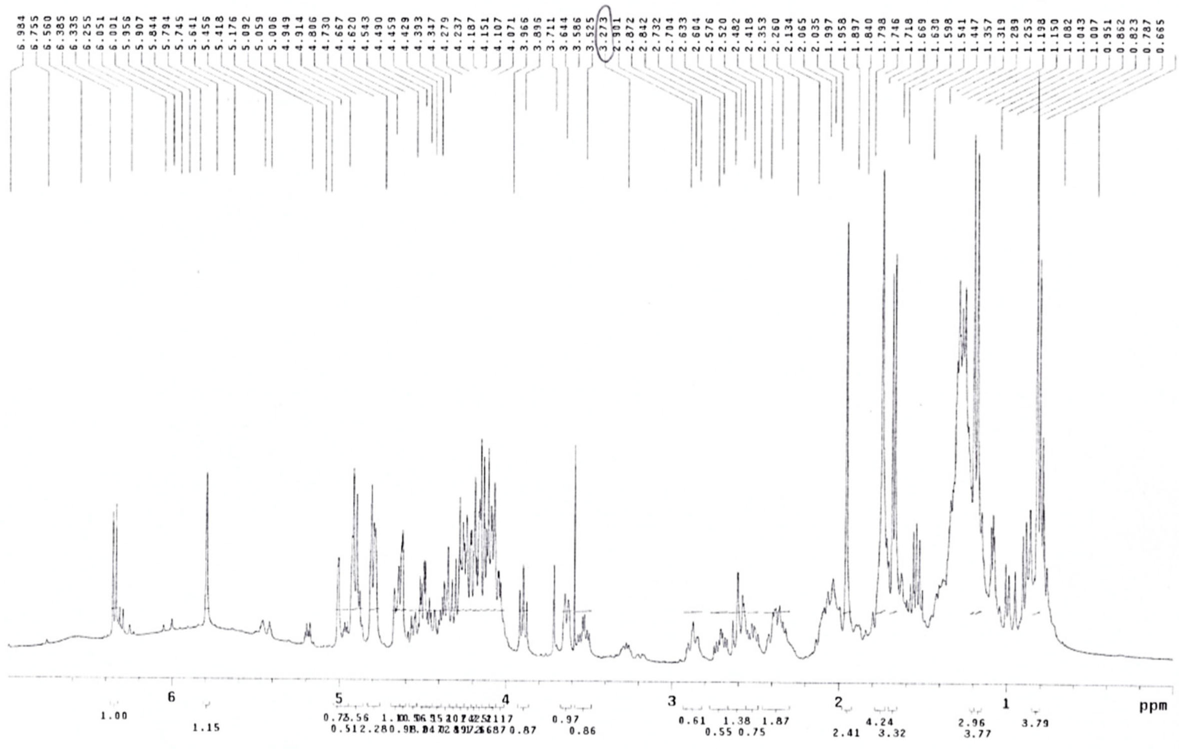

^{13}C NMR of Acanthosessilioside K

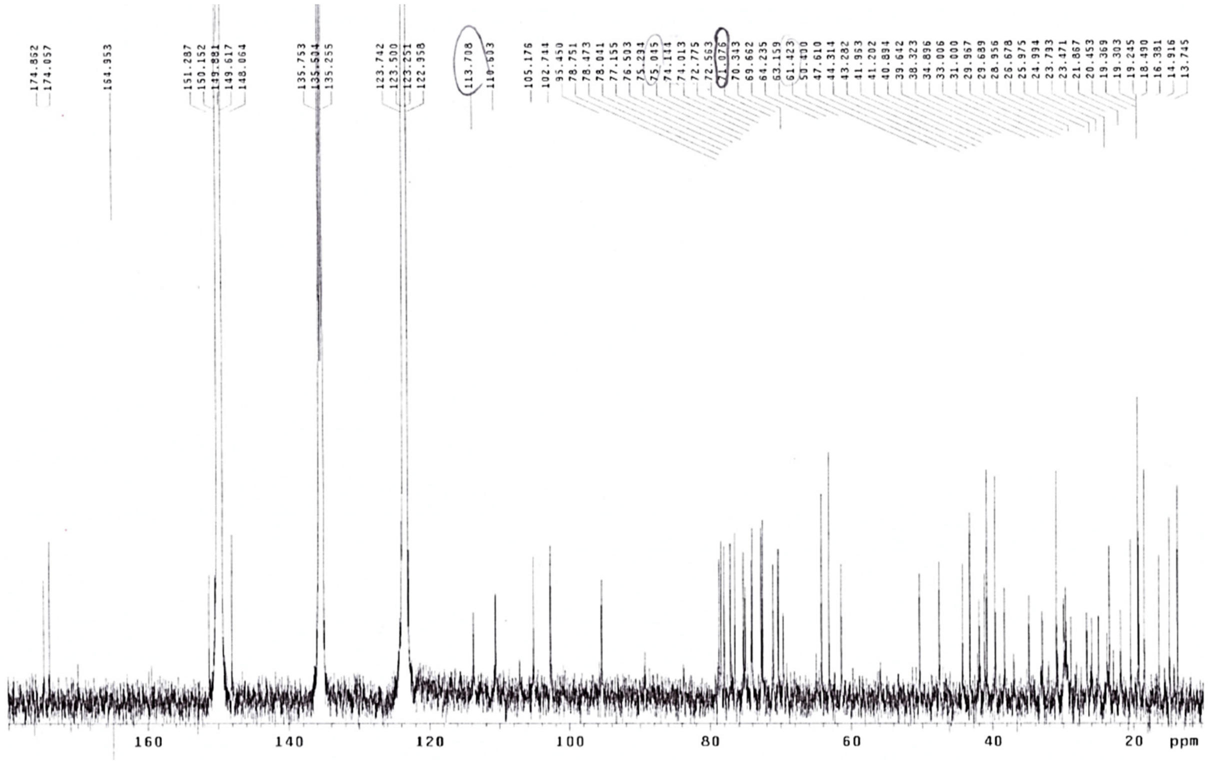

Qtrap/MSMS of Acanthosessilioside K

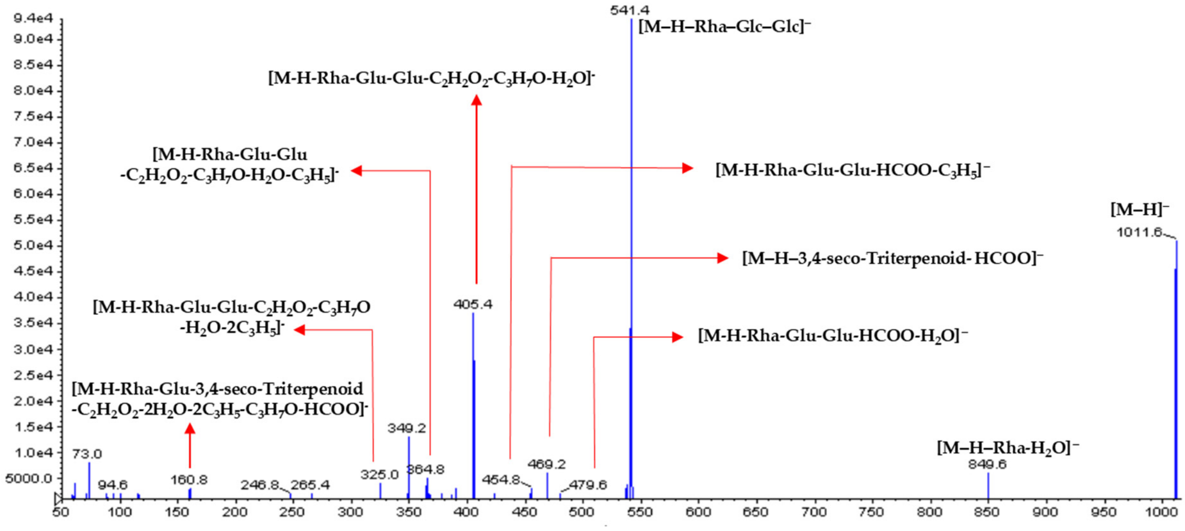

HRESIMS of Acanthosessilioside K

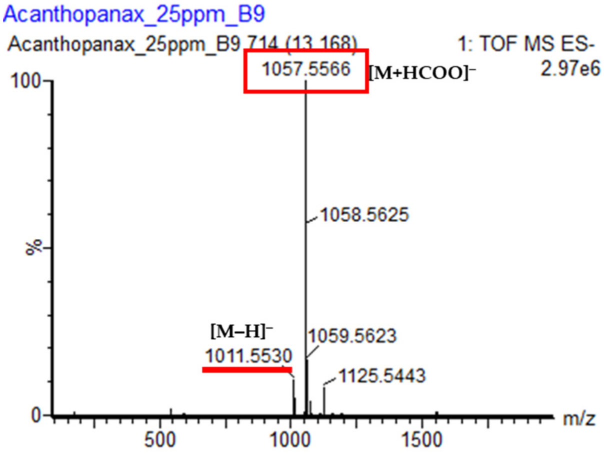

S6: ^{1}H, ^{13}C NMR, Qtrap/MSMS, and HRESIMS of Acanthosessilioside L

^{1}H NMR of Acanthosessilioside L

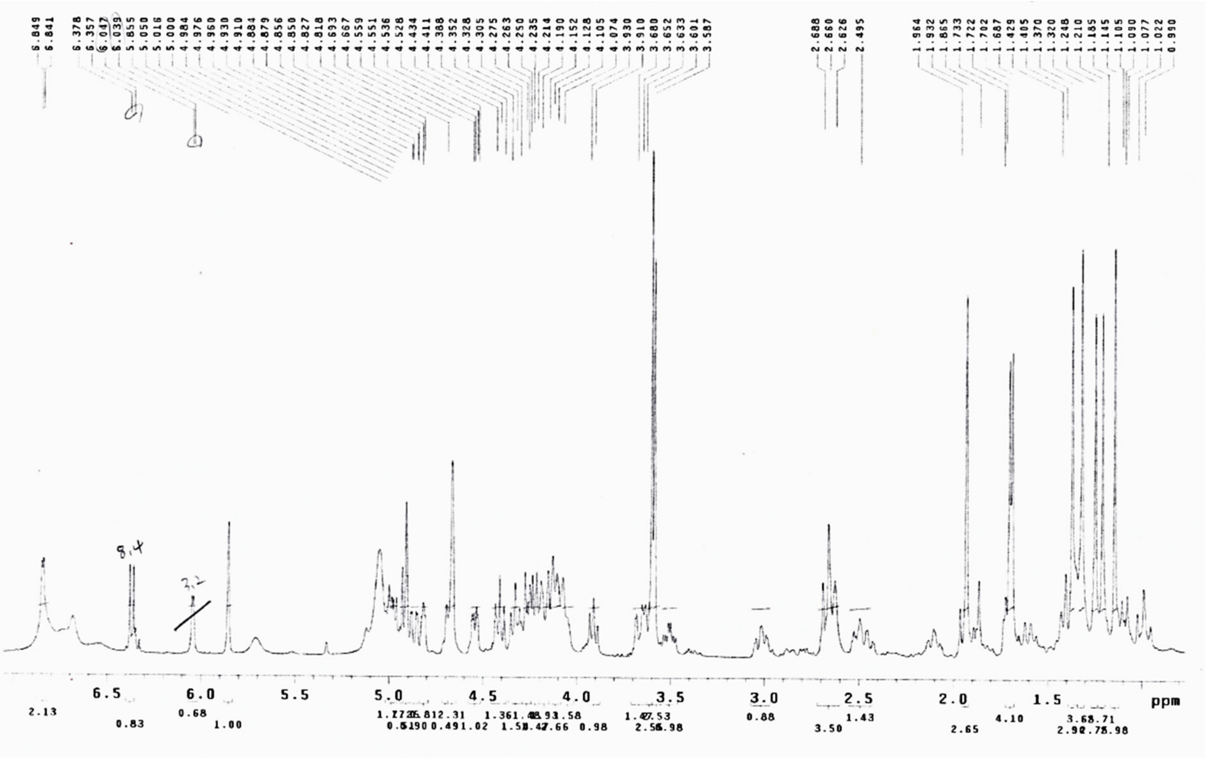

^{13}C NMR of Acanthosessilioside L

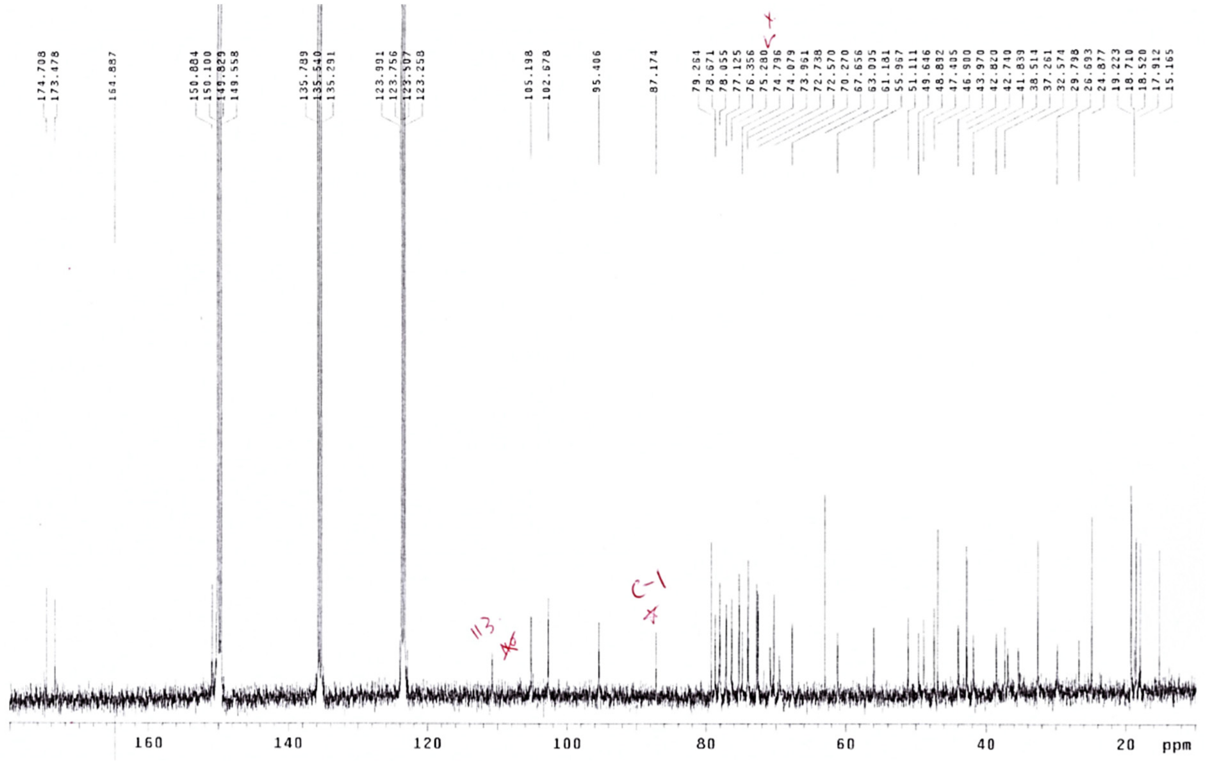

Qtrap/MSMS of Acanthosessilioside L

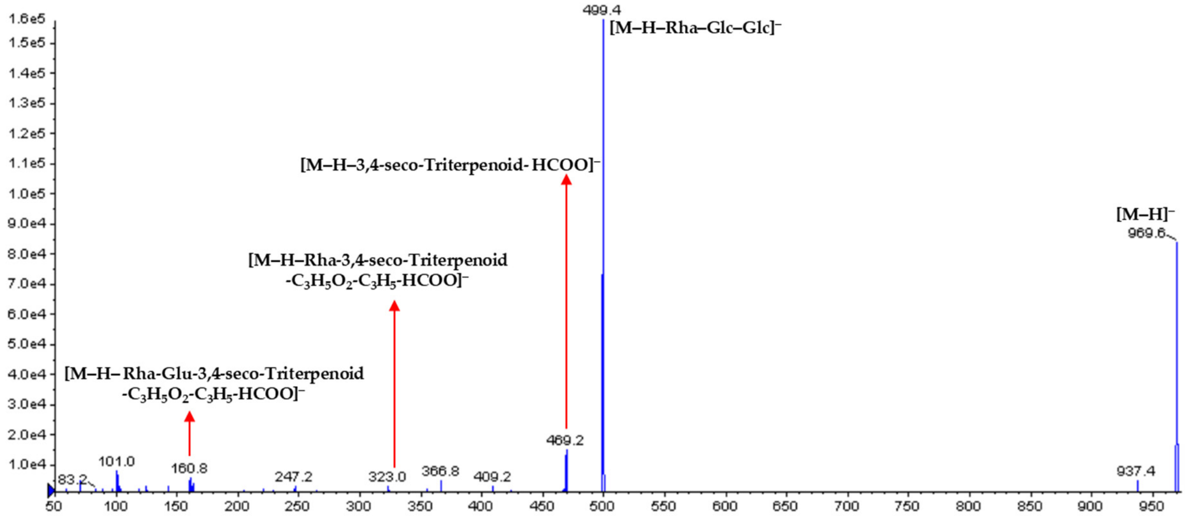

HRESIMS of Acanthosessilioside L

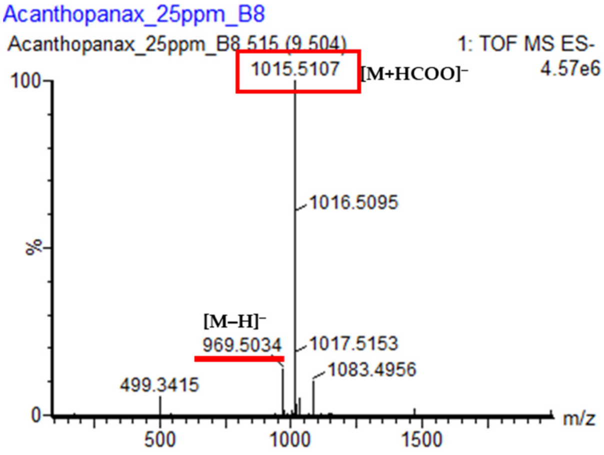

S7: ^{1}H, ^{13}C NMR, Qtrap/MSMS, and HRESIMS of Acanthosessilioside M

^{1}H NMR of Acanthosessilioside M

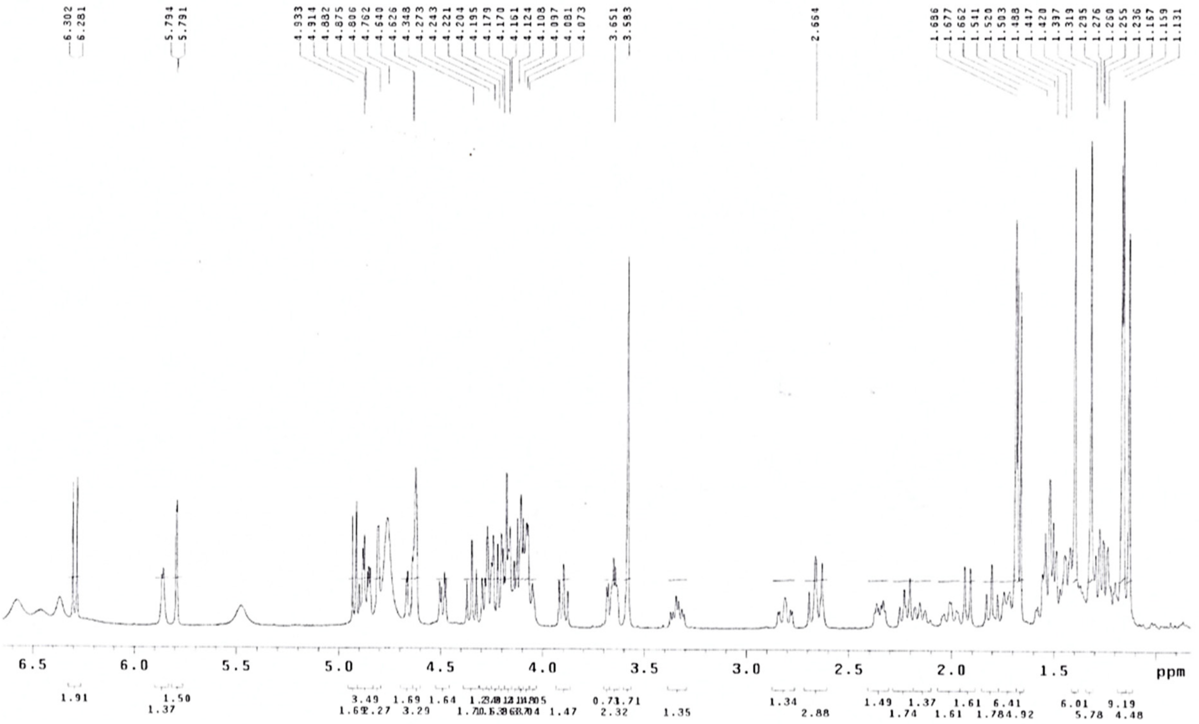

^{13}C NMR of Acanthosessilioside M

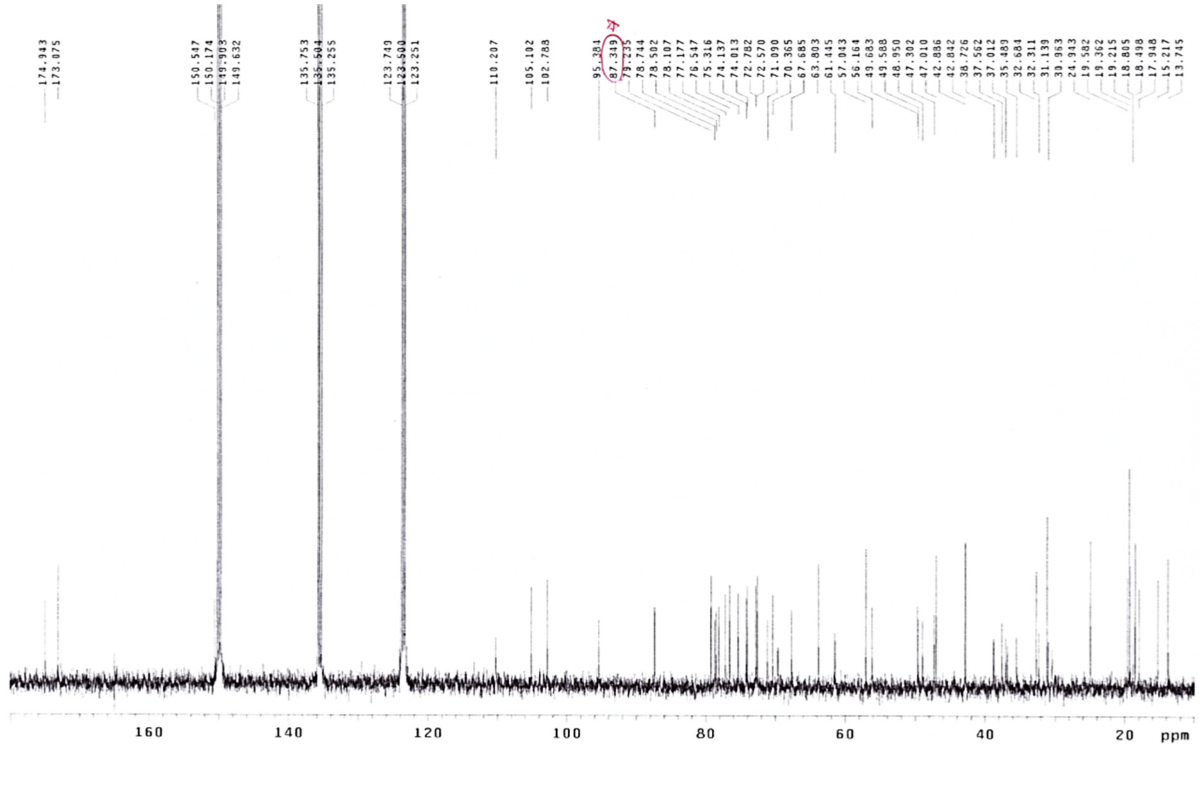

Qtrap/MSMS of Acanthosessilioside M

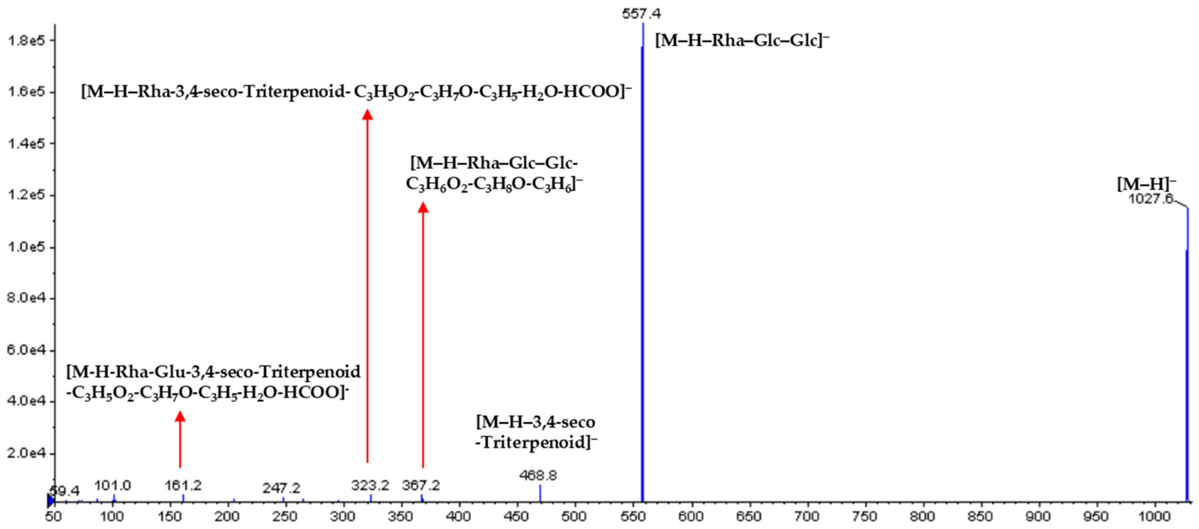

HRESIMS of Acanthosessilioside M

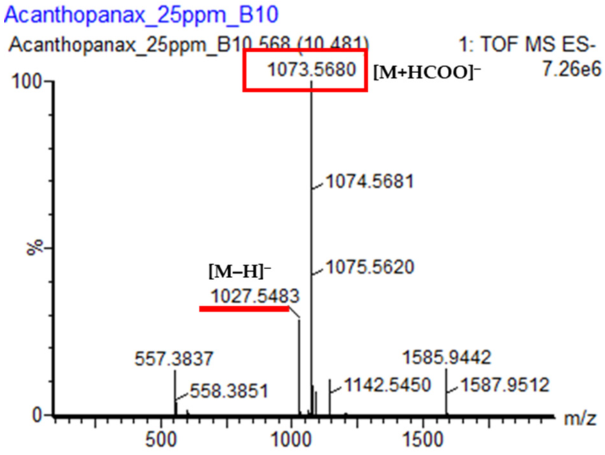

S8: ^{1}H, ^{13}C NMR, Qtrap/MSMS, and HRESIMS of Acanthosessilioside N

^{1}H NMR of Acanthosessilioside N

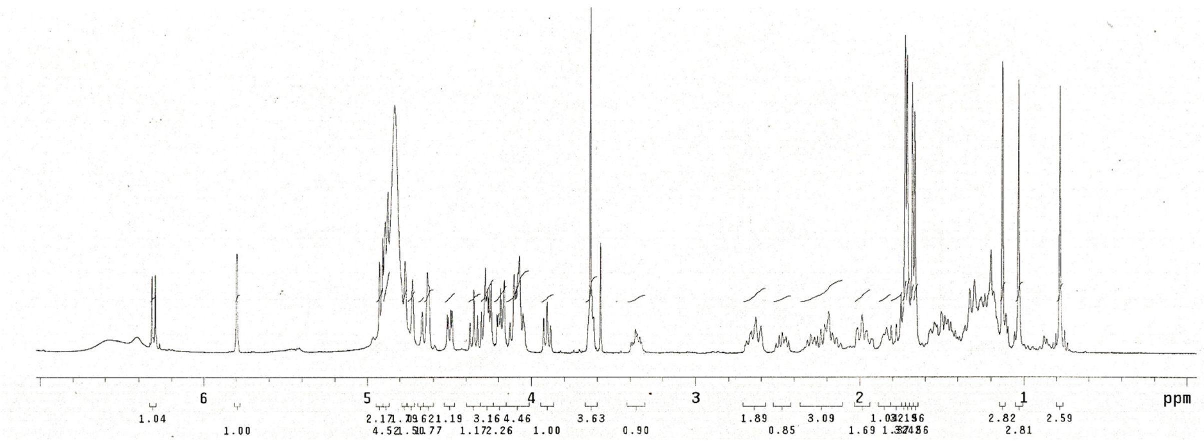

^{13}C NMR of Acanthosessilioside N

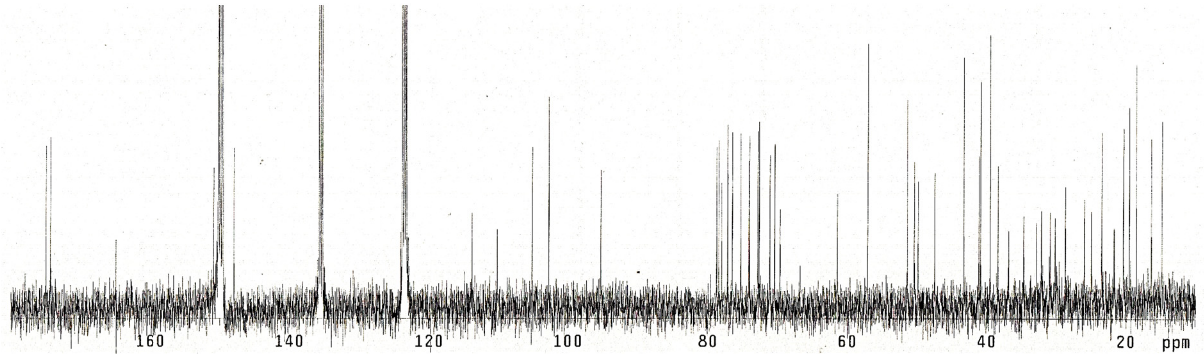

Qtrap/MSMS of Acanthosessilioside N

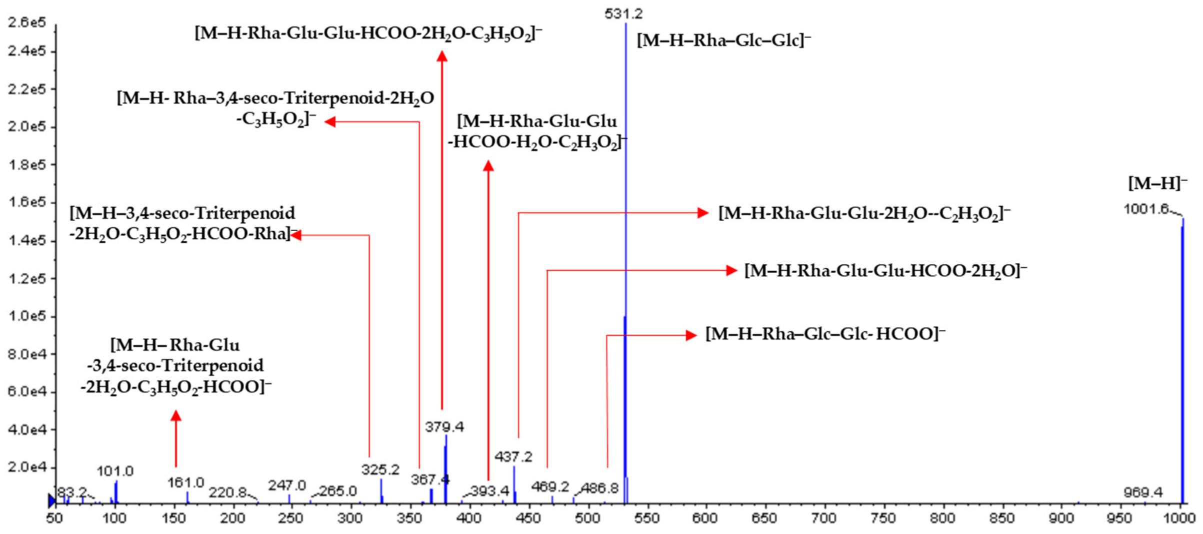

HRESIMS of Acanthosessilioside N

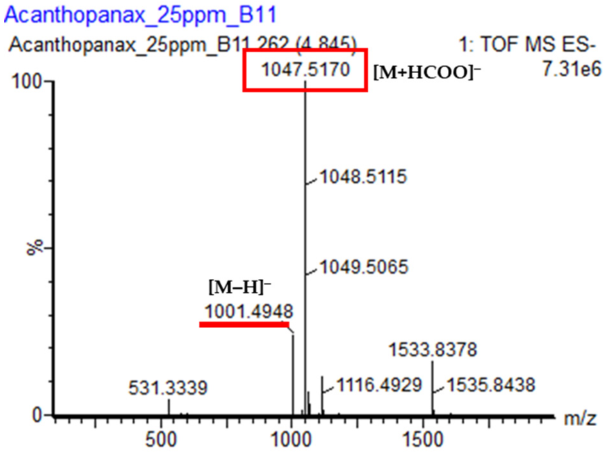

S9: ^{1}H, ^{13}C NMR, Qtrap/MSMS, and HRESIMS of Acanthosessilioside O

^{1}H NMR of Acanthosessilioside O

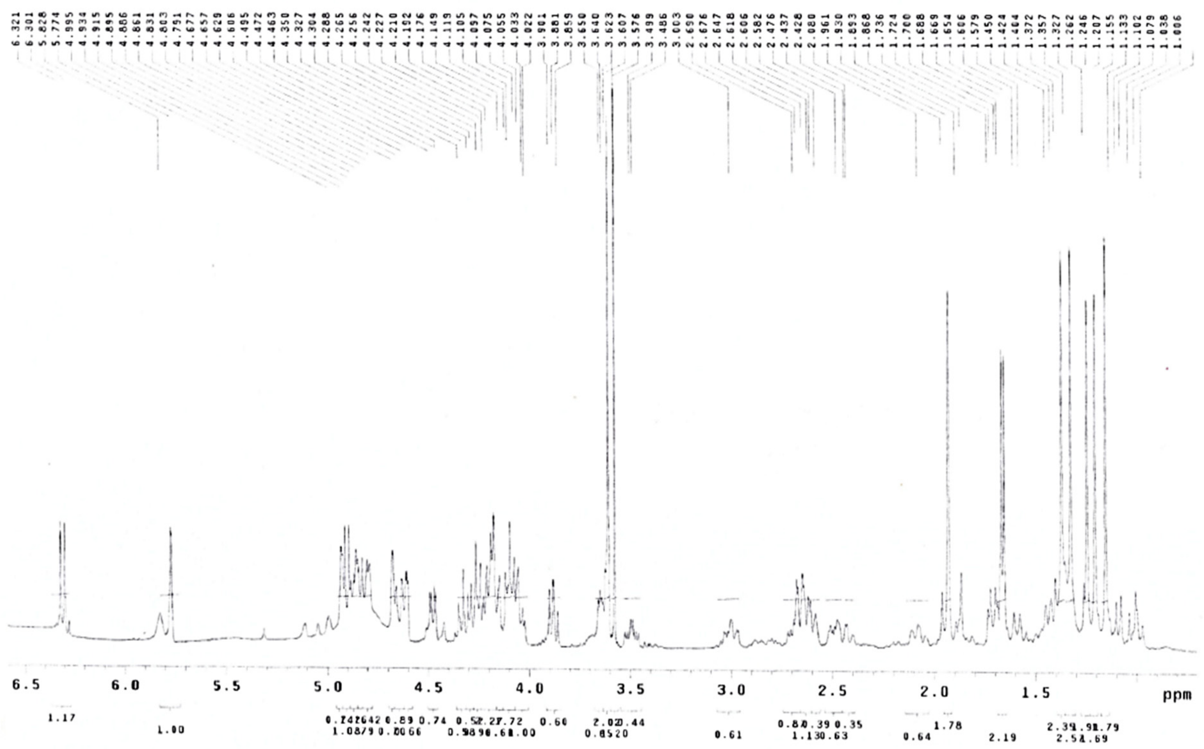

^{13}C NMR of Acanthosessilioside O

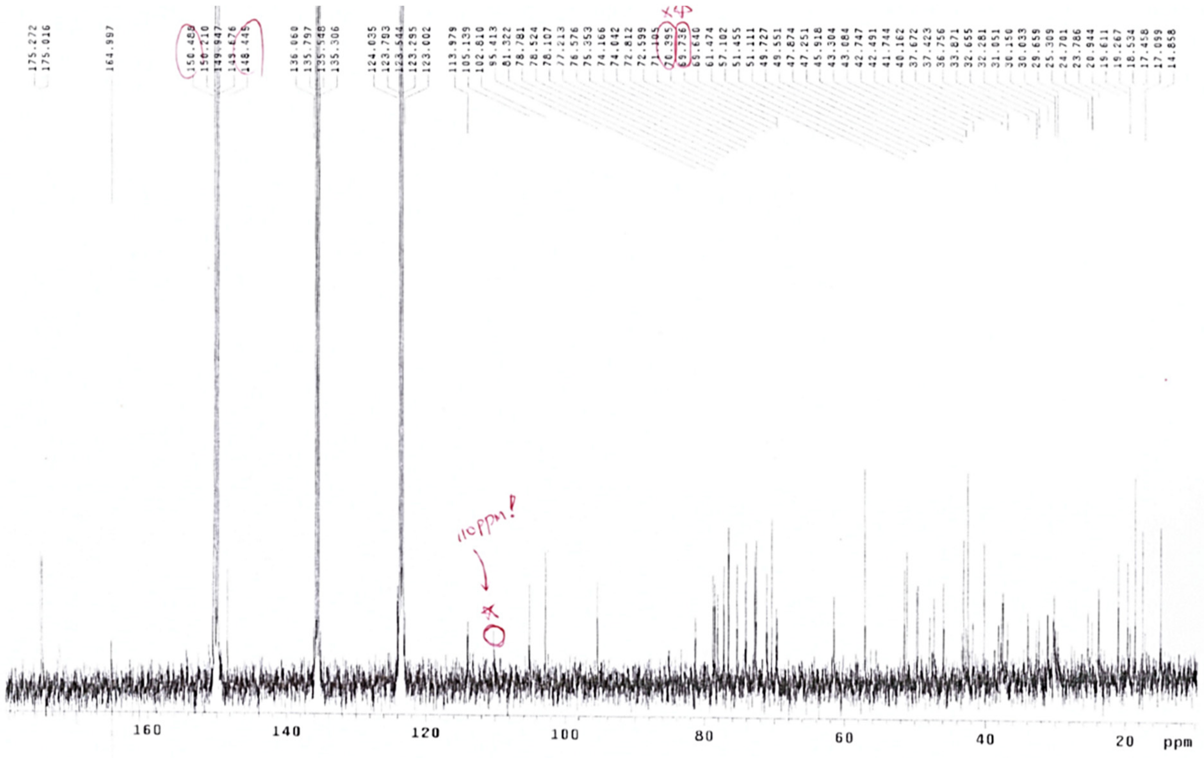

Qtrap/MSMS of Acanthosessilioside O

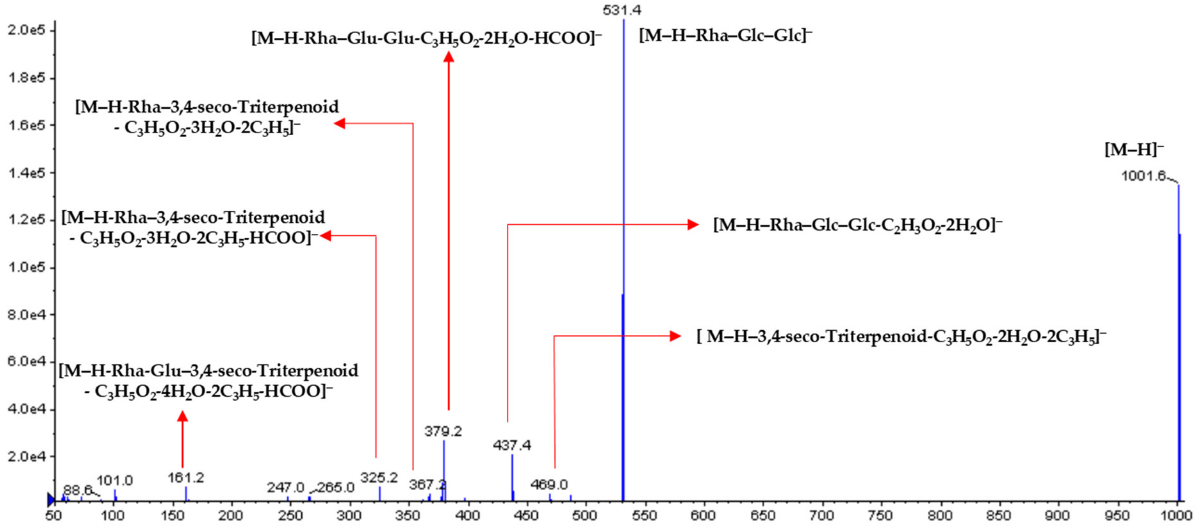

HRESIMS of Acanthosessilioside O

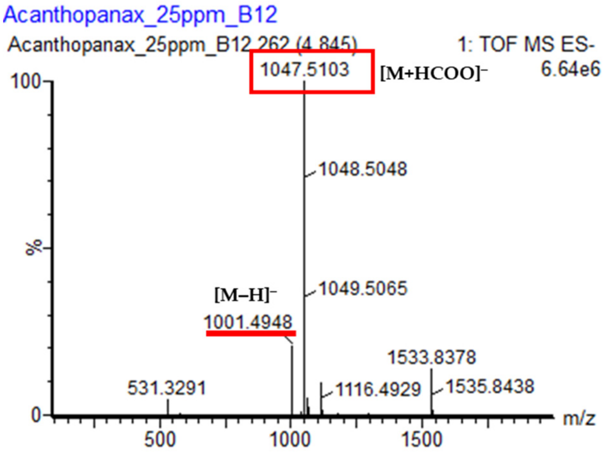

Supplement: Supplementary file 1 [file antioxidants-10-01334-s001.zip › antioxidants-1252573-supplementary.pdf]
